# Adjunctive Treatment with Rhodiola Crenulata in Patients with Chronic Obstructive Pulmonary Disease – A Randomized Placebo Controlled Double Blind Clinical Trial

**DOI:** 10.1371/journal.pone.0128142

**Published:** 2015-06-22

**Authors:** Ming-Lung Chuang, Tzu-Chin Wu, Yau-Tung Wang, Yau-Chen Wang, Thomas C.-Y. Tsao, James Cheng-Chung Wei, Chia-Yin Chen, I-Feng Lin

**Affiliations:** 1 Division of Pulmonary Medicine and Department of Critical Care Medicine, Chung Shan Medical University Hospital, Taichung, Taiwan; 2 School of Medicine, Chung Shan Medical University, Taichung, Taiwan; 3 Chinese Medicine Clinical Trial Center, Chung Shan Medical University Hospital, Taichung, Taiwan; 4 Institute of Medicine, Chung Shan Medical University, Taichung, Taiwan; 5 Graduate Institute of Integrated Medicine, China Medical University, Taichung, Taiwan; 6 Institute and Department of Public Health, National Yang Ming University, Taipei, Taiwan; University of Ottawa, CANADA

## Abstract

Chronic obstructive pulmonary disease (COPD) is a low grade systemic inflammatory disease characterized by dyspnea and exercise intolerance even under standard therapy. *Rhodiola crenulata* (RC) has been shown to exert anti-inflammatory effects and to enhance exercise endurance, thereby having the potential to treat COPD. In this 12-week, randomized, double-blind, placebo-controlled clinical trial, 57 patients with stable moderate-to-severe COPD aged 70±8.8 years were given RC (250 mg twice/day) (n=38) or a placebo (250 mg twice/day) (n=19) in addition to their standard regimen. There were no significant differences in anthropometrics, quality of life, lung function, six-minute walk and incremental exercise tests between the two groups at enrollment. Over the 12 weeks, RC was well tolerated, significantly reduced triceps skin thickness (Δ=-1 mm, p=.04), change of FEV1 (4.5%, p=.03), and improved workload (Δ=10%, p=.01); although there were no significant differences in these factors between the two groups. However, there were significant between-group differences in tidal volume and ventilation-CO_2_-output ratio at peak exercise (both p=.05), which were significantly related to peak work rate (both p<.0001). RC tended to protect against acute exacerbation of COPD (p=.1) but not other measurements. RC did not improve the six-minute walk test distance but significantly improved tidal breathing and ventilation efficiency, most likely through improvements in work rate. Further studies with a larger patient population are needed in order to confirm these findings.

**Trial Registration:**

ClinicalTrials.gov number NCT02242461

## Introduction

Chronic obstructive pulmonary disease (COPD) is one of the top ten causes of death in Taiwan [1], and is projected to be one of the top three causes of death worldwide by 2020. Most patients present with dyspnea and exercise intolerance due to deranged lungs and impaired peripheral oxygenation to the skeletomuscular system and mitochondria [2]. The mainstay of treatment is an inhaled combination of bronchodilators and corticosteroids [3], and pulmonary rehabilitation [4] may also be beneficial. However, COPD is low-grade on-going systemic inflammation making it difficult to control with local therapy [5,6].

Corticosteroid and roflumilast treatment has been reported to result in a substantial number of side-effects [7,8], whereas N-acetylcysteine and infliximab, an anti-TNF-α agent, have no effect. *Rhodiola rosea* L. extract (RR) contains p-tyrosol, salidroside, rosavin, pyridine, rhodiosin and rhodionin [9,10], and has been reported to exert anti-inflammatory and anti-oxidative effects [11–13]. RR has also been reported to enhance endurance [14], physical and mental performance in healthy subjects [15], and the quality of life in patients with hypothyroidism [16] with minimal side-effects after long-term use [17]. However, it has never been used for the management of patients with COPD [18]. Even though *Rhodiola crenulata* (RC) has been shown to have a lesser effect on the ATP content in the mitochondria of Sprague-Dawley rats [19], RC combined with Ginkgo biloba has been shown to enhance endurance performance in healthy volunteers [14]. We hypothesized that adding RC for 12 weeks to a standard regimen for COPD patients may improve their quality of life and exercise tolerance.

## Materials and Methods

### Study Design

This was a 12-week randomized, double-blind, placebo-controlled clinical trial conducted at Chung Shan Medical University Hospital (CSH-2012-C-023).

### Subjects

We enrolled and followed patients with moderate to severe COPD from the pulmonary division of a university hospital in Taiwan from January 1, 2012 to December 31, 2012. The local institutional review board of Chung Shan Medical University Hospital approved this study (CSHRP 11144). This study is registered at ClinicalTrials.gov (NCT02242461). The authors did not establish requirements with regards to registering for the study before enrolment of participants started. The authors confirmed that all related trials for this study were registered. The patients were aged 40–80 years, abstained from cigarette smoking or maintained a low consumption, had no acute exacerbation of COPD, were stable for 1 month or longer in terms of clinical condition, adhered to the medication for COPD suggested by the GOLD guidelines [4], and did not partake in any exercise training program. The patients who took prednisolone >10 mg per day, had uncontrolled diabetes mellitus, uremia, chronic heart failure, cerebrovascular disease, uncontrolled anemia, late-stage malignant diseases, other acute illness, or those taking any systemic anti-inflammatory agents were excluded.

### Ingredients

Each capsule contained 250 mg RC with 1.99 mg salidroside per capsule (standard: >1.2 mg per capsule) or a placebo, and the capsules were produced by a cGMP pharmaceutical company (Chuang Song Zong Pharmaceutical Co., Ltd., Kaohsiung, Taiwan).

### Study protocol

All eligible subjects with moderate to severe COPD were enrolled after signing informed consent forms. The participants were randomly allocated to the study group or the control group on a 2:1 ratio using a computer-generated randomization schedule. The sample size was estimated to be 60 based on calculations with the primary outcome, and the block randomization size was six. To ensure concealment, the pharmacist put the experimental drug or placebo into each envelope in advance, and the envelopes were numbered in sequence according to the allocation assignment. The pharmaceutical company manufactured the appearance of the capsules of the experimental drug and placebo to be identical. The participants, care providers, and those assessing outcomes were blinded during the whole study period. The reason for the 2:1 ratio in subject number was based on our previous experience of clinical trial studies, in which most of the subjects would prefer not to attend the trial should they be assigned to the placebo group. The study group took a RC capsule (250 mg) twice per day for 12 weeks, and the control group took the placebo of the same dose for the same duration. The following data were recorded after randomization: demographics, anthropometrics, dyspnea scored with the Borg scale, chronic respiratory disease questionnaire (CRDQ) [20], modified medical research council (mMRC) and oxygen-cost diagram (OCD) [21], and cough and sputum evaluated with the COPD assessment test (CAT) [22]. In addition, composite indices including BODE [23], a multidimensional 10-point scale of body mass index, severity of airflow obstruction, dyspnea rated with the mMRC, and exercise capacity evaluated with the Six-Minute Walk Distance (6MWD) [23], quality of life evaluated with the St. George Respiratory Questionnaire (SGRQ) [24], anxiety depression with the Hospital Anxiety Depression Scale (HADS), and sleepiness with the Epworth sleepiness scale were evaluated. Blood tests including complete blood cell count and biochemical analysis, lung function, Six-Minute Walk Test (6MWT), Cardiopulmonary Exercise Test (CPET), and COPD acute exacerbations (defined as more coughing with more yellow sputum than usual or the need for more prednisolone for rescue or the need for antibiotics in cases of possible infection or for more dyspnea which would require the subject to visit the emergency room or to be hospitalized) were also evaluated [4]. Side-effects and serious adverse effects of RC and the placebo were reported by each patient every time they occurred, and were followed-up monthly by the study nurses. At the end of the 12-week study, all of the subjects underwent the same measurements as at entry to the study (baseline). Each subject revisited the out-patient clinic of the hospital every 4 weeks. The predetermined primary outcome measure was differences in the 6MWD between baseline and 12 weeks. The predetermined secondary outcome measures were differences in the quality of life, blood tests, and factors related to the CPET. The full trial protocol can be accessed via (S1 File. The protocol).

### Measurements

#### Anthropometric measurements

Triceps skin thickness was measured with calipers, and mid-upper arm circumference was measured with a measuring tape. Both measurements were taken midway between the tip of the olecranon process and the acromion process, in the midline of the posterior surface of the extended dominant arm. All measurements were made three times by a trained study nurse and the middle value was recorded for analysis.

#### OCD, mMRC and BODE

Please refer to (S2 File. List of abbreviations and symptom scaling system).

#### Quality of life

Selected questionnaires were used to evaluate the patients’ quality of life, including the CRDQ dyspnea component [20], SGRQ (S3 File. Permission to use the SGRQ)[24], HADS, baseline dyspnea index, and transitional dyspnea index [25].

#### Blood tests

Complete blood cell count and high-sensitivity C-reactive protein (hs-CRP, rate turbidimetry) were measured. Biochemical analysis including alanine transaminase, aspartate aminotransferase, creatinine, and blood urea nitrogen were assessed using an automatic analyzer (ARCO, Biotechnica Instruments, Italy) at the indicated times.

#### Pulmonary function test

Forced expired volume in one second (FEV_1_), total lung capacity, and residual volume were measured with a pressure-sensitive body plethysmograph (MasterScreen Body, Carefusion, Leibnizstrasse, Wuerzburg, Germany) at body temperature, ambient atmospheric pressure, and when fully saturated. The best of three technically satisfactory readings was used [26]. All of the lung function data were obtained after inhaling 400 μg of fenoterol HCl. The diffusing capacity for carbon monoxide was measured using the single-breath technique. Direct maximum voluntary ventilation was performed, and simple volume calibration was conducted with a 3-L syringe before each test.

#### Six-minute Walk Test

The walking tests were conducted in a temperature-controlled 20-meter corridor. The 6MWT was conducted with verbal encouragement as per the American Thoracic Society recommendations [27]. SpO_2_ and pulse rate readings were continuously determined by pulse oximetry (N-595, Nellcor/Covidien, Boulder, Colorado). A minimal clinically significant difference of SpO_2_ was defined as a decrease ≥3% from the start of the exercise. Each patient performed the 6MWT twice, with >30 minutes rest in between. The longest distance walked was recorded in meters.

#### Maximum cardiopulmonary exercise test

For details of the exercise protocol and the conduction of CPET, please refer to the studies by Chuang and Lin [2] and the ATS/ACCP Statement on cardiopulmonary exercise testing [2,28]. Workload, heart rate, oxyhemoglobin saturation (8000AA Adult Finger Clip, Nonin Medical, Plymouth, MN, USA), oxygen uptake ($\dot{\text{V}}$ O_2_ (ml/min)), CO_2_ output ($\dot{\text{V}}$ CO_2_ (ml/min)), minute ventilation, and blood pressure were measured (MasterScreen CPX, CareFusion, Leibnizstrasse, Wuerzburg, Germany). The exercise data were averaged and reported every 15 seconds. Only data from the exercises where the patients exerted themselves well were retained for analysis [22]. The $\dot{\text{V}}$ O_2peak_ achieved by the patients was the symptom-limited highest recorded point and designated $\dot{\text{V}}$ O_2peak_ or $\dot{\text{V}}$ O_2max_.

### Statistical Analysis

Intent-to-treat analysis and per-protocol analysis were used in this study. The intent-to-treat analysis was used between the study group and the placebo group. The multiple imputation procedure with the Markov chain Monte Carlo method was used to impute missing values of the primary endpoint, walking distance. FVC, FEV_1_, diffusing capacity for carbon monoxide, OCD, and Borg’s score were simultaneously considered, and the imputed values were constrained at positive values. Five sets of imputed data were generated, and the results were combined using SAS MI and MIANALYZE procedures. Per-protocol analysis was used between the two groups for differences between pre- and post-intervention of the CPET data. Data were summarized as mean ± standard deviation (SD) or median (interquartile). Taking into account the correlations between pre- and post-intervention within the same individual, a linear mixed model was used to compare the means in each outcome variable between pre- and post-intervention, and to test these pre-post differences between the study and placebo groups. For each outcome variable, the comparisons were planned a priori, and *p* values were obtained from the same model by establishing appropriate dummy variables and interactions between time (post- or pre-intervention) and group (treatment or placebo). For non-normal data, the Mann-Whitney test was used. The chi-square test or Fisher’s exact test was used to compare the proportion of categorical variables between the two groups. A *p* value of less than .05 was considered to be statistically significant, and a *p* value of less than .1 but more than .05 was considered as being a trend. Statistical procedures were performed using the SAS software package version 9.3 (SAS Institute Inc., Cary, NC) and Microcal Origin v 4.0 (Northampton, MA, USA).

## Results

### Enrollment and Outcomes of the Study Protocol

**Fig 1:**
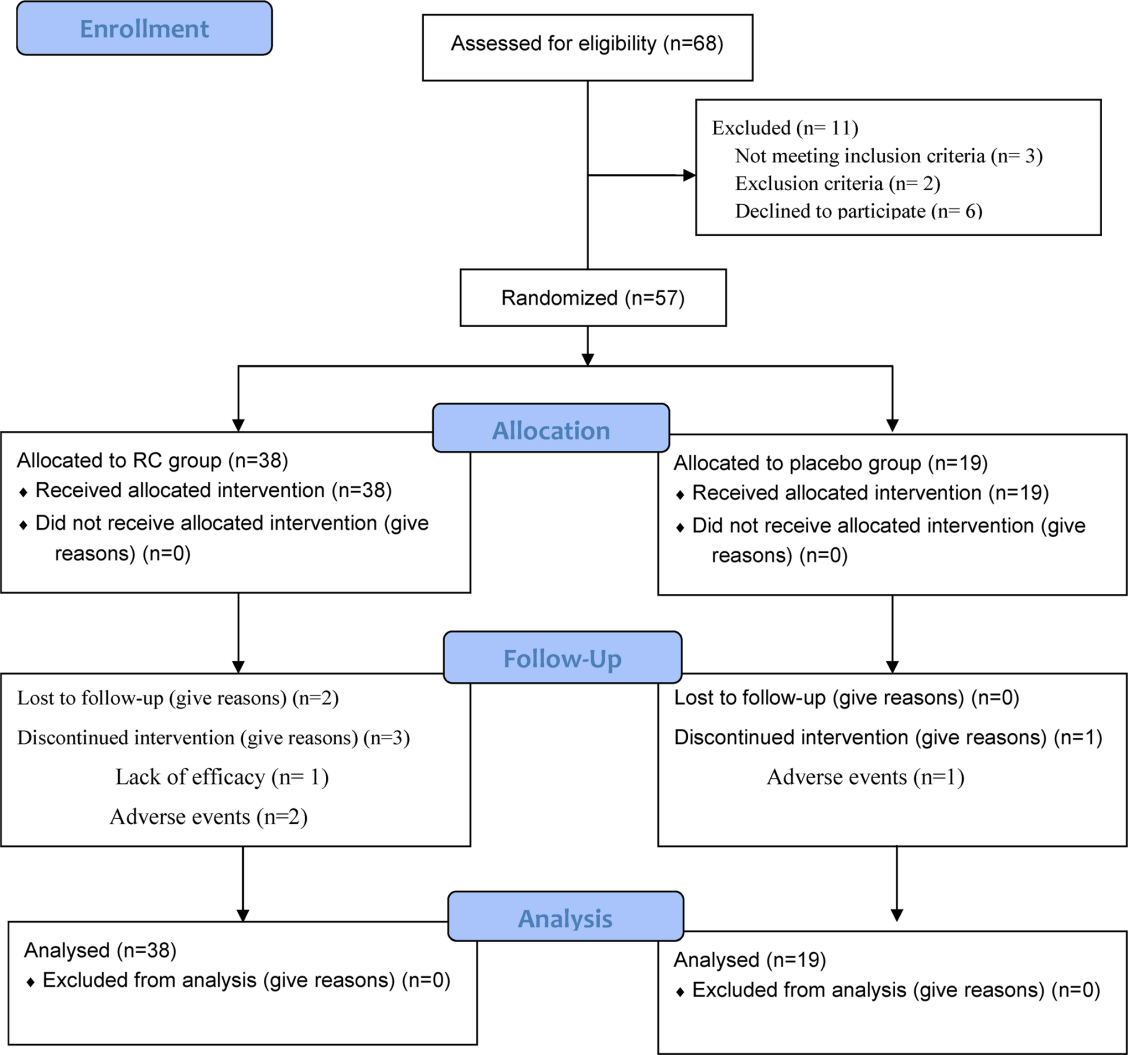
Flow Diagram. A total of 68 patients with chronic obstructive pulmonary disease were screened. Eleven patients were excluded, and 57 subjects were randomly allocated to the study group or control group with a 2:1 ratio. This 2:1 ratio was based on our previous experience with regards to clinical trial studies. Please refer to the text.

Of the 68 patients screened (Fig 1), 57 were enrolled, and 38 of these 57 patients were randomized into the study group and 19 into the placebo group (Table 1) (Please also see S4 File. CONSORT 2010 Checklist (2)). Five subjects dropped out from the study group and one from the placebo group, however there was no difference in dropout rate between the two groups (Table 2, p = .65).

**Table 1:** Patient demographics and baseline characteristics.

Characteristics	*Rhodiola*	Placebo
	(n = 38)	(n = 19)
Age, years	70.1±7.6	69.8±11.2
Gender: M/F	37/1	19/0
Cigarette, pack-years	42.9±19.8	44±25.9
BMI, kg/m^2^	23.9±3.6	25.9±4.7
Triceps skinfold thickness, mm	17±7.4	19.7±5.4
Mid upper arm circumference, cm	27.5±3.1	29.1±3.9
Systolic BP, mm Hg	136±17	138±14
Diastolic BP, mm Hg	79±10	72±10
Medications used at entry, n (%)		
SAMA+SABA	11	6
LAMA	17	8
LABA	6	4
LABA+ICS	22	13
LABA+LAMA+ICS	12	5
Xanthines	23	12
Antihistamine	9	4
FVC, L	2.93±.78	2.89±.6
FEV_1_, L	1.46±.55	1.58±.42
FEV_1_% pred, %	60.6±18.8	64.3±10.3
FEV_1_ reversibility % pred, %	9.6±9.8	3±5.7
Oxygen-cost diagram, mm (100–0)	66±12	71±10
modified Medical Research Council, AU (0–4)	1±.8	1±.9
Borg score, AU (0–10)	.7±1	.5±1.1
COPD assessment test, AU (0–40)	10.8±7	10.1±8.5
Baseline dyspnea index score, AU (12–0)	7.2±1.9	7.8±2.1
CRDQ (35–5)	27.4±4.7	28.6±3.7
SGRQ (0–100)		
Total score	35.1±16.6	31.7±17.8
Symptoms score	36.3±18.9	31.5±16.7
Activity score	48.5±22.7	45.3±25.6
Impact score	27.2±16.7	23.2±17.1
Hospital anxiety depression score (0–42)	6.1±5.3	6.3±5.6
Anxiety (0–21)	2.6±2.7	2.3±2.5
Depression (0–21)	3.4±3	4±3.7
Epworth sleepiness scale score (0–24)	6.3±4.2	5.6±3.1
BODE (0–10)	1.8±2.2	1.1±1.2
Six-minute walking distance, meter	445.7±135.5	421.9±66.8
Oxygen uptake_peak_ % pred, %	74±15	67±10
White blood cell, /mm^3^	7,196±2,168	7,652±2,055
Hemoglobin, gm/dL	14.6±1.4	14.7±1.7
Platelet, /mm^3^	219k±57k	211k±62k
Creatinine, mg/dL	1±.2	1±.3
hs-CRP, mg/dL	.48±.61	.86±1.66

Mean ± SD or median (IQR). BMI: body mass index; ICS: inhaled corticosteroids; SAMA/LAMA: short-acting/long-acting muscarinic antagonist; SABA/LABA: short-acting/long-acting β_2_-agonist; CAT: COPD assessment test score; CRDQ: chronic respiratory disease questionnaire; SGRQ: St George’s respiratory questionnaire; BODE: body mass index, airway obstruction severity, dyspnea graded with the mMRC, and 6-minute walk distance; hs-CRP: high sensitivity C-reactive protein. (the 1^st^ number - the 2^nd^ number): in the parenthesis, from the 1^st^ number to the 2^nd^ number indicate a better status to a worse status; Anxiety or depression score ≤7: no anxiety, 8–10: possible, ≥ 11: definitely.

**Table 2:** Dropout rate and adverse effects of *Rhodiola crenulata*.

Groups	Rhodiola (n = 38)	Placebo (n = 19)	p
Dropout, n (%)	5 (13.1)	1 (5.3)	0.65
Acute exacerbation, n	1 (2.6)	0 (0)	
Poor appetite/upset, n	1 (2.6)	1 (5.3)	
Loss to follow-up, n	2 (5.3)	0 (0)	
No effect, n	1 (2.6)	0 (0)	
Acute exacerbation	1 (2.6)	3 (15.8)	0.1
Common cold	13 (34.2)	4 (21)	0.3
Cough	7 (18.4)	2 (10.5)	0.7
Dyspnea	1 (2.6)	1 (5.3)	1
Upper airway dryness	1 (2.6)	0 (0)	1
Functional GI problems	5 (13.1)	1 (5.3)	0.65
Dizziness and giddiness	1 (2.6)	0 (0)	1
Gout	0 (0)	1 (5.3)	0.33
Severe adverse effect	0 (0)	0 (0)	-
Any side effect	21 (55.3)	11 (57.9)	0.85

For definitions of acute exacerbation, please refer to text. GI: gastrointestinal. Chi square or Fisher’s exact test was used for comparisons.

### Baseline data of the patients

There were no significant differences in baseline demographics, anthropometrics, quality of life, blood tests, lung function, 6-minute walk distance, maximum oxygen uptake, and medication use between the two groups (Table 1).

### Primary Outcome: 6MWD

**Fig 2:**
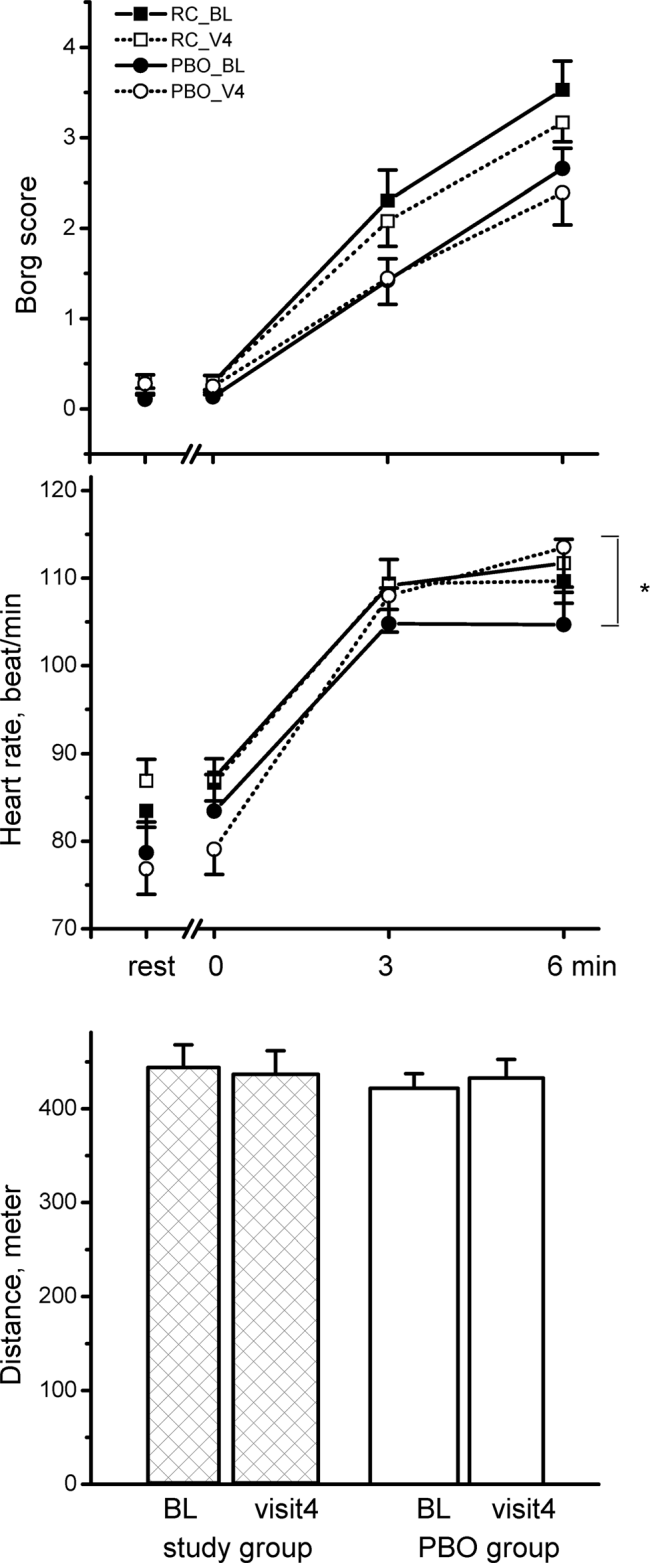
Differences in the selected variables from the six-minute walk test between the *Rhodiola crenulata* group and placebo (PBO) group over the 12-week treatment period. *comparison within group: p < .05, comparisons between groups: all p >.05. Solid square symbol: baseline test of the study group, open square symbol: test at 12 weeks of the study group, solid circle symbol: baseline test of the placebo group, open circle symbol: test at 12 weeks of the placebo group, bars: standard error.

Over the 12 weeks, RC did not improve 6MWD (Fig 2, Δ = -7.5 meters, p = .23), and there was no change in heart rate at the end of the 6MWT (Fig 2, Δ = -.4 b/min, p = .6). The placebo also did not improve 6MWD but significantly increased heart rate at the end of the 6MWT (Δ = 9 b/min, p = .03). There were no significant differences in 6MWD over the 12-week study period between the two groups (Fig 2, all p>.05).

### Secondary Outcomes

**Fig 3:**
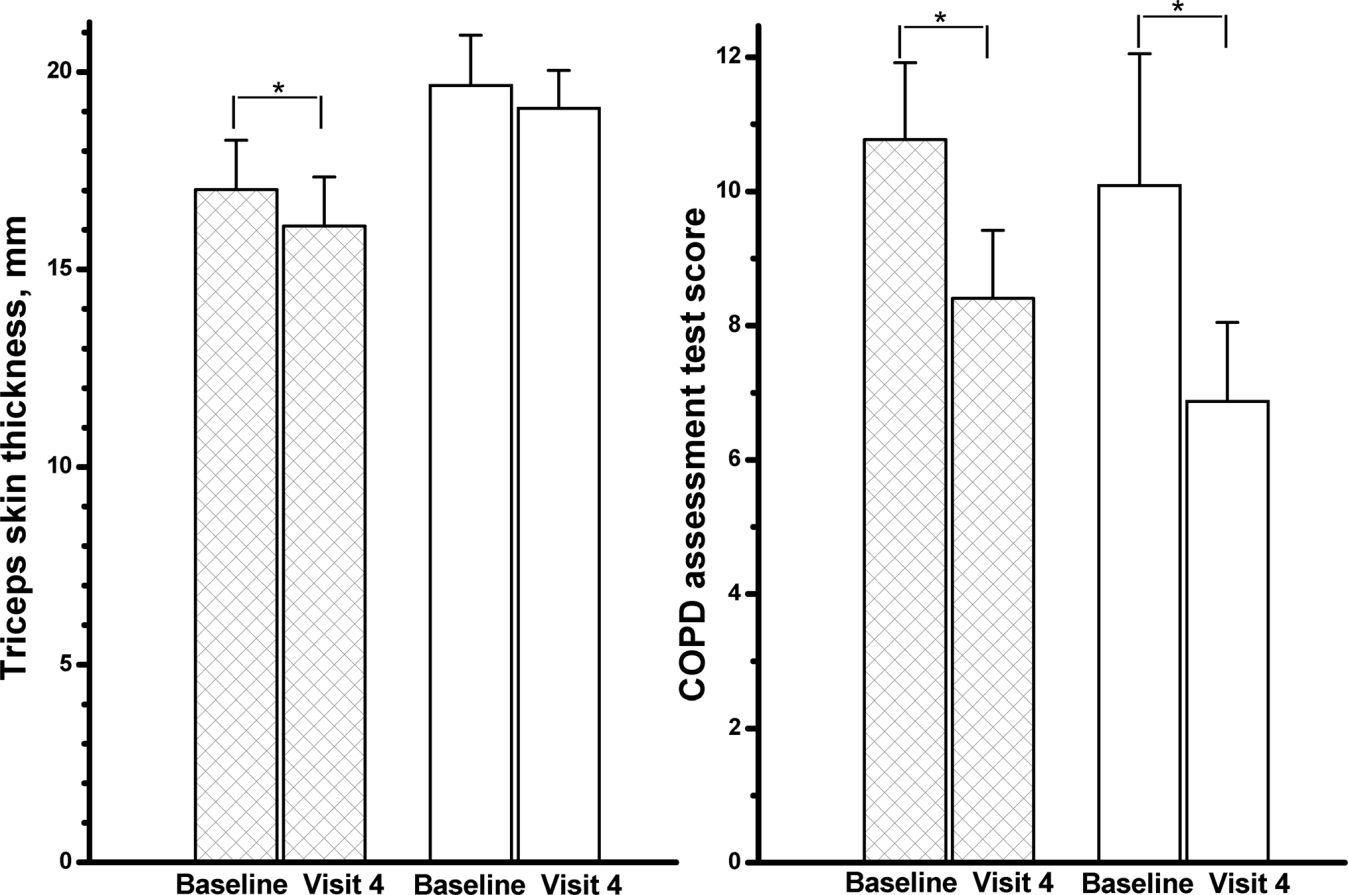
Differences in triceps skin thickness and COPD assessment test score between the *Rhodiola crenulata* group (hatched columns) and placebo (PBO) group (open columns) over the 12-week treatment period. Visit 4 = at 12 weeks, bars: standard error. *comparisons within group: p < .05, comparisons between groups: both p >.05.

**Fig 4:**
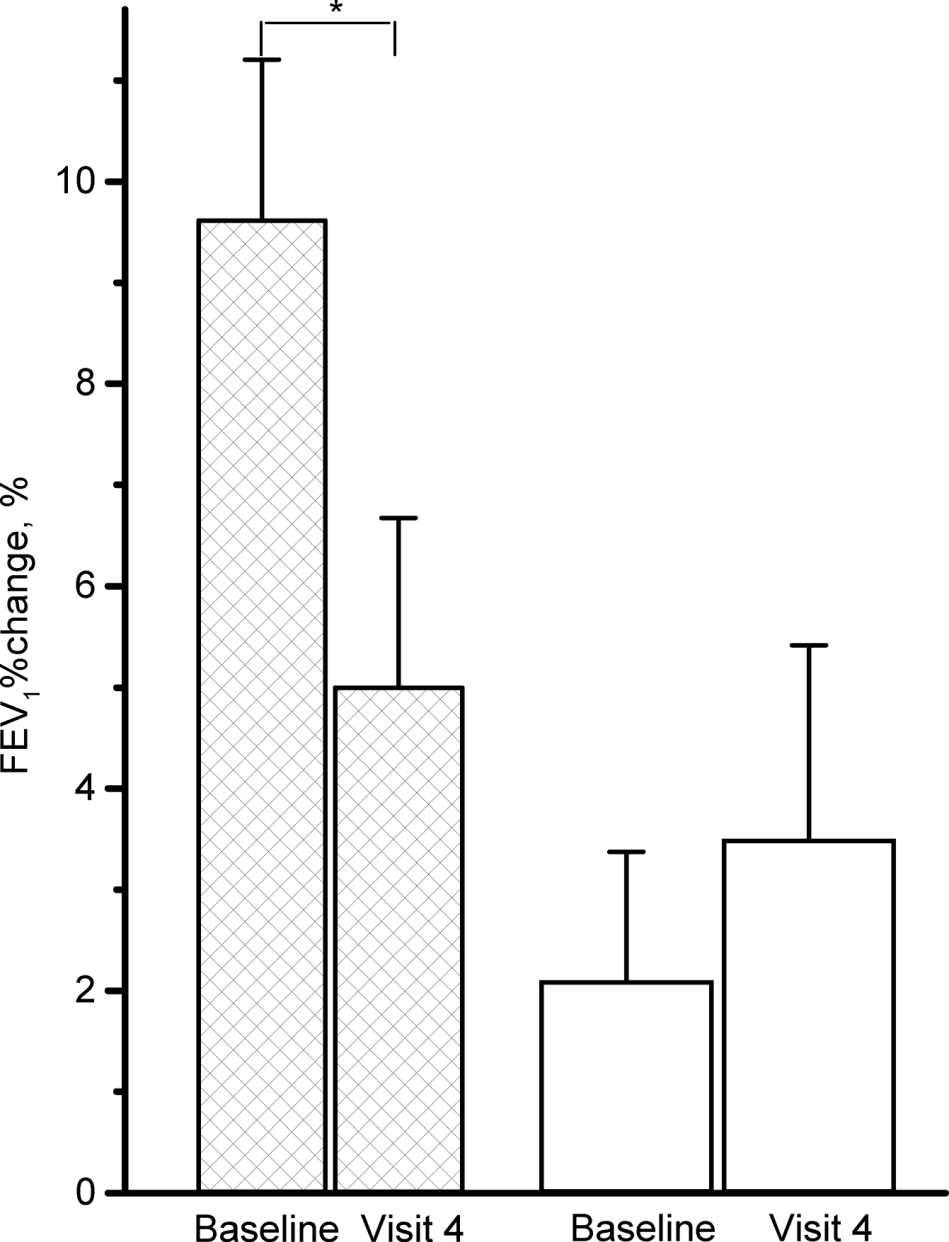
Differences in the selected variables from lung function test between the *Rhodiola crenulata* group (hatched columns) and placebo (PBO) group (open columns) over the 12-week treatment period. FEV_1_%change = % change of forced expired volume in one second (FEV_1_) after inhaling fenoterol HCl, bars: standard error. *comparisons within group: p < .05, comparison between groups: p >.05.

Over the 12-week study period, RC significantly decreased triceps skin thickness (Fig 3, Δ = -1 mm, p = .04), CAT score (Δ = -2.4, p = .04), change in FEV_1_ after inhaling fenoterol HCl (Fig 4, Δ = -4.5%, p = .03) and hs-CRP (Δ = .21 mg/dL, p = .38). The placebo also improved CAT score (Δ = -3.2, p = .03). There were no significant differences in the secondary outcomes over the 12-week study period between the two groups (Figs 3 and 4, all p>.05).

**Fig 5:**
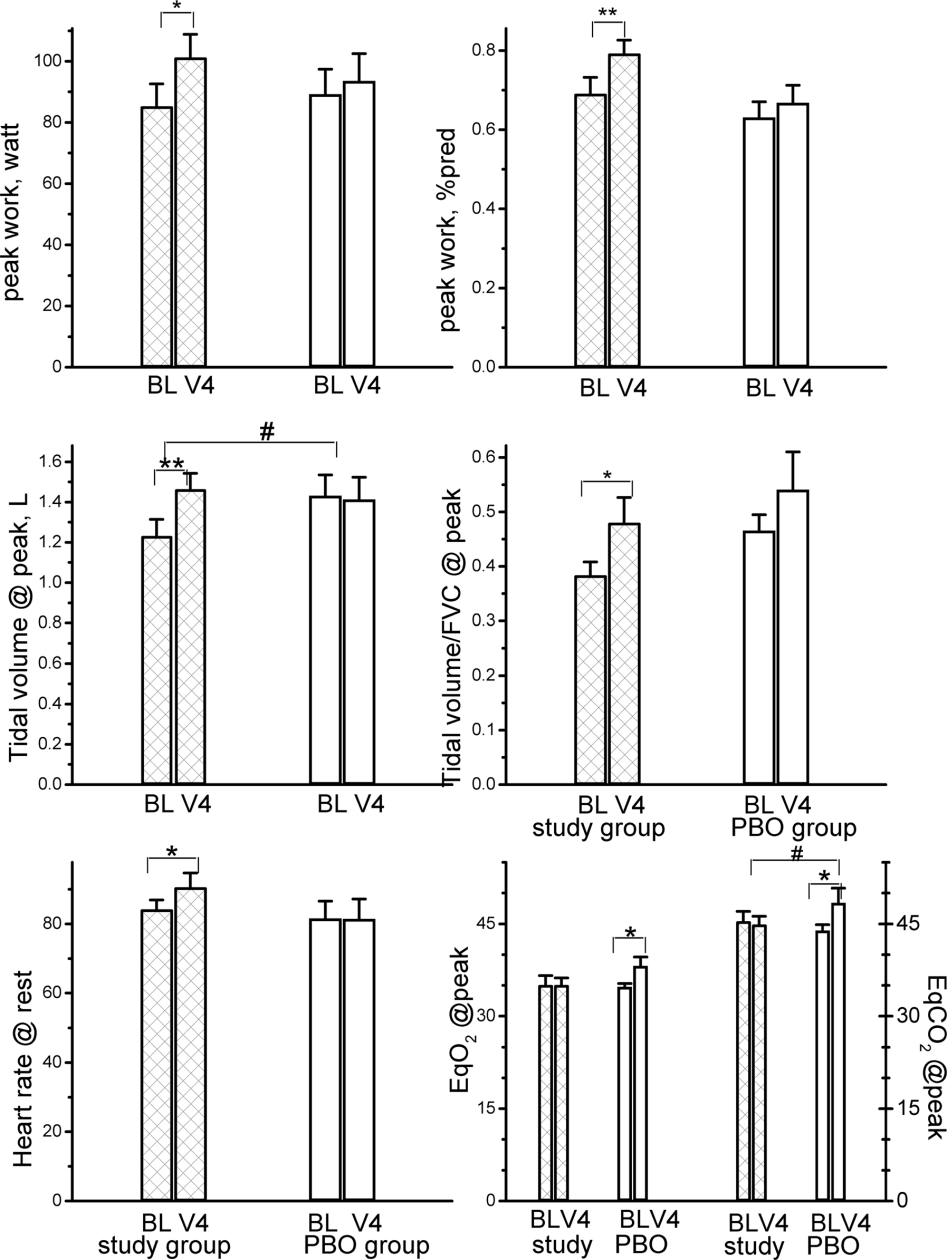
Differences in the selected variables from maximum incremental exercise test between the *Rhodiola crenulata* group and placebo (PBO) group over the 12-week treatment period. BL = baseline, V4 = visit at 12 weeks, EqO_2_ and EqCO_2_ = ventilatory equivalents for O_2_ uptake and CO_2_ output, bars: standard error. *comparisons within group: p < .05, ** comparisons within group: p < .01, ^#^comparisons between groups: p < .05.

**Fig 6:**
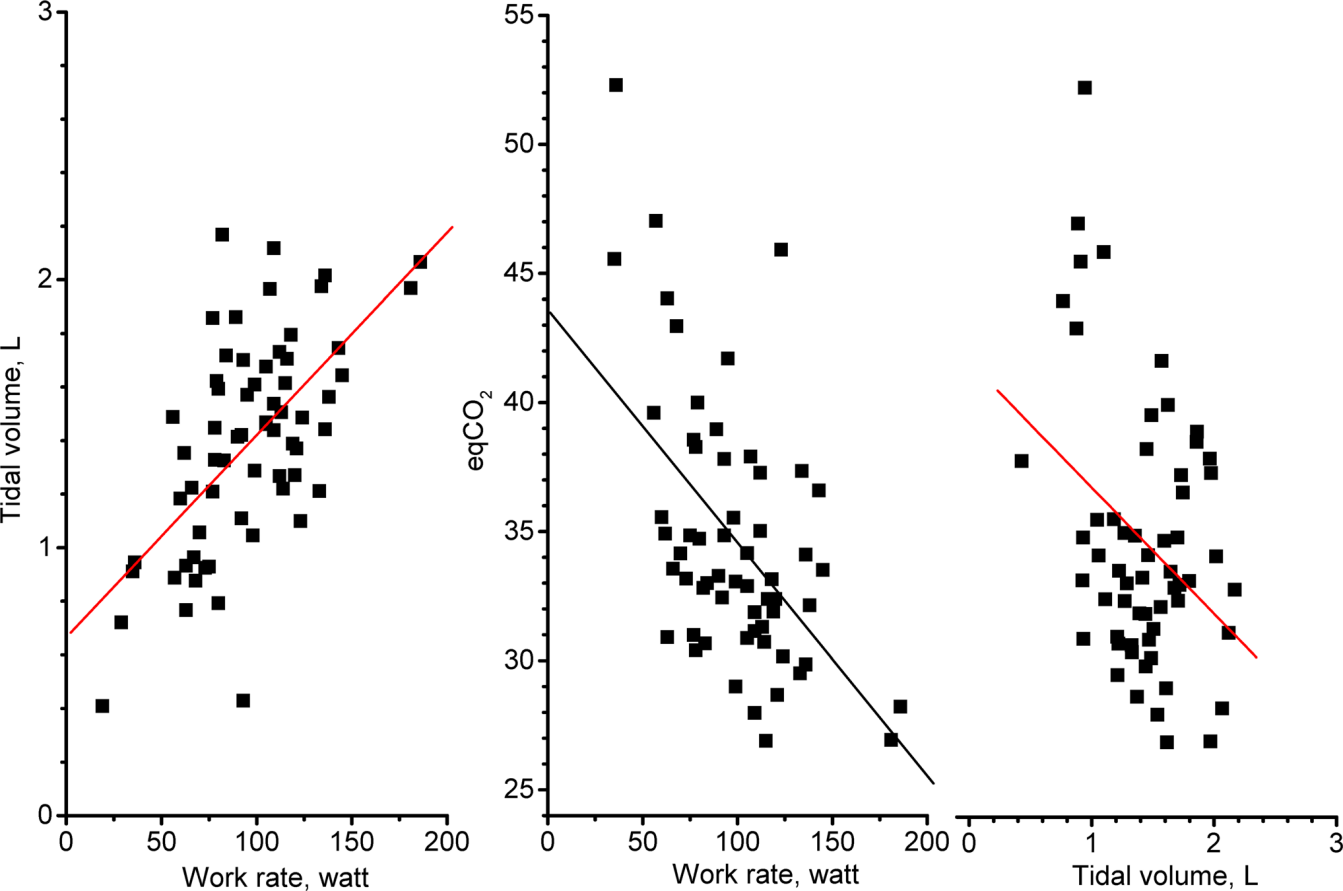
Relationship between tidal volume, ventilatory equivalent for CO_2_ output (eqCO_2_), and work rate at peak exercise. Left panel: Relationship between tidal volume and work rate at peak exercise in all patients over the treatment period (r = 0.61, p < .0001); Middle panel: Relationship between eqCO_2_ and work rate at peak exercise in all patients over the treatment period (r = -.54, p < .0001); Right panel: Relationship between eqCO_2_ and tidal volume at peak exercise in all patients over the treatment period (r = -.35, p < .01).

Over the 12-week study period, RC significantly improved the maximum workload from 85.2 (69% predicted) to 101.2 (79% predicted) watts (Fig 5, upper panels, both p < .05), and also modestly increased heart rate at rest (from 84 to 90 b/min, p = .02). RC also improved tidal volume at peak exercise (p < .01) and maintained the ventilatory equivalent for O_2_ uptake at peak exercise ($\dot{\text{V}}$_E_/$\dot{\text{V}}$ O_2 peak_) and ventilatory equivalent for CO_2_ output at peak exercise ($\dot{\text{V}}$_E_/$\dot{\text{V}}$ CO_2 peak_), although there was no significant change over the 12 weeks (p = .38 and .53, respectively). In contrast, the placebo significantly elevated the $\dot{\text{V}}$_E_/$\dot{\text{V}}$ O_2_ and $\dot{\text{V}}$_E_/$\dot{\text{V}}$ CO_2_ (p = .05 and .04, respectively). Between the two groups, RC improved the tidal volume and $\dot{\text{V}}$_E_/$\dot{\text{V}}$ CO_2_ at peak exercise (both p = .05), and both improvements were related to increases in workload (Fig 6, both p < .0001).

### Adverse Events and COPD acute Exacerbations

Although side-effects were encountered in 55.3% of the RC group, they were also encountered in 57.9% of the placebo group (Table 2, p = .85). Over the 12-week study period, there was a trend of less frequent COPD acute exacerbations in the RC group (p = .1).

## Discussion

This study demonstrates that the adjunctive use of RC for patients with COPD is well tolerated and improves tidal breathing and ventilation efficiency during incremental exercise, but does not have an impact on anthropometrics, quality of life, lung function, six-minute walk distance, and hs-CRP. RC treatment also led to a trend of less frequent COPD acute exacerbations. To the best of our knowledge, this is the first clinical trial of RC on the clinical performance of patients with COPD.

### Primary outcome

A recent meta-analysis of principal plants including Astragalus membranaceus, Panax ginseng and Cordyceps sinensis not including RC or RR supported their effects on the 6MWD or BODE, but suggested that interpretation of the results should be made cautiously due to variations in Chinese herbal medicines and the standard therapy for COPD and methodological weakness in the studies [18]. We did not identify benefits of RC use on the 6MWD or BODE in this study. RC may have prevented a higher heart rate at the end of the 6MWT (Fig 2, Δ = -.4 b/min, p = .6), however there was no significant difference compared with placebo treatment (p = .16) over the treatment period. It is worth noting that this may be due to type II errors.

### Secondary outcome: CPET

A combination of *Rhodiola crenulata* and *Gingko biloba* was reported to improve maximum oxygen uptake and protect against fatigue in young healthy volunteers over a 7-week treatment period [14]. RR was also reported to improve peak oxygen uptake (n = 12, Δ = 2 ml/min/kg) in a 2-day trial but not in a 4-week trial [29], suggesting the acute effect on exercise endurance capacity in young healthy subjects. Another clinical trial crossover study (n = 18) also confirmed the acute effect of RR in improving endurance exercise performance by reducing the perception of effort [30]. In contrast, other clinical trial studies including a 2-week trial for cyclists [31], a 4-week trial for marathon runners [32], and a 4-week trial for trained male athletes [33] confirmed no effects of chronic use on exercise performance.

In this study, RC did not increase maximum oxygen uptake but it did increase maximum workload by 10% (p < .01) over the 12-week treatment period. This suggests that the regular use of RC may improve strenuous physical effort by improving the ergogenic effect but not by improving cardiovascular function. However, there was no difference in improvements of maximum workload between the two groups (p = .2) even though the placebo did not improve the maximum workload.

Tidal volume was significantly improved by RC over the treatment period (Fig 5, between-group difference, p = .05). RC also maintained $\dot{\text{V}}$_E_/$\dot{\text{V}}$ CO_2 peak_, an indicator of ventilation efficiency, over the 12-week period; however, the placebo elevated $\dot{\text{V}}$_E_/$\dot{\text{V}}$ CO_2 peak_ (between-group difference, p = .05). This was related to a higher workload performance at peak exercise, thereby improving tidal volume and ventilation efficiency (Fig 6, r = .61 and-.54, both p < .0001) rather than improving lung function (all p >.05).

### Other secondary outcomes

RR has been reported to possibly reduce levels of plasma free fatty acids [33]. RR plus *Citrus aurantium* has been reported to act on central monoamine pathways and to have the potential to reduce 30% of visceral fat [34]. In this study, RC significantly reduced the triceps skin thickness (Δ = -1 mm, p = .04) over the treatment period. However, the difference in the between-group comparison was insignificant (p = .7).

RR has been reported to exert anti-inflammatory and anti-oxidative effects in animals [11–13] and humans [35], however our results cannot confirm these findings as the hs-CRP level did not improve (p = .38). This may be because both groups had a hs-CRP level of <1.55 mg/dL (Table 1) and a threshold of normal values [36], so that there was no room for improvement over the treatment period. Another possible reason is that metabolic syndrome, physical activity, and GOLD stages, independent predictors for the elevation of hs-CRP in patients with COPD [37], were excluded from this study or were similar between the two groups (both p >.05).

RR has been reported to enhance physical, mental [15], cognitive, and adaptogenic performance among healthy subjects, reduce burnout in patients with fatigue syndrome [9], and improve depression in patients with hypothyroidism [16] or mild to moderate depression [38]. However, the efficacy of RR in previous studies has been criticized due to flaws in the study design [39]. In this 12-week placebo-controlled, double-blind and randomized study, RC did not improve CAT score, daily dyspnea score and activities, CRDQ, SGRQ, HADS, or daytime sleepiness scores.

RC tended to protect the COPD patients against acute exacerbations (p = .1) but not mild cough, dyspnea or common cold (all p>.3). A few side-effects were noted over the 12-week treatment period, consistent with reports regarding the long-term use of RR [17,38,40]. Although both the study drug and placebo produced side-effects, the side-effects were mild and the incidence of side-effects were similar, suggesting that RC was well tolerated in this study.

### Study Limitations

The trial registration of this study was not carried out before initiating the study as we were not aware this was required for a dietary supplement study. In addition, the study population was small and subject to type II errors, and the duration of the study was short at 12 weeks; however, this is much longer than that of previous studies [14,29,31,33,34] and still provides additional information. The reason why other secondary outcomes did not improve in this study may also be because we did not use the same questionnaires as reported previously [40]. Negative findings (insignificant differences) should be interpreted with caution, and further studies with a larger sample size are warranted. The insignificant differences may have been caused by a larger standard deviation of the 6MWD in this study compared to previous studies [41], and an insufficient sample size due to the inappropriate selection of the sample size calculation method in this study. Given the small sample size, however, many significant differences were detected between pre- and post-treatment in each group, and some significant differences were detected in changes between the two groups over the treatment period.

Despite randomly allocating the subjects to the study or placebo group, a single block size for randomization is a threat to concealment of allocation. However, the thorough design of this double blind trial should diminish this threat to the lowest level. Knol et al reported that *p*-values reported in baseline tables of randomized controlled trials are inappropriate [42]; however, we consider the small scale of the population in this study and the 2:1 ratio of allocation of the subjects may contribute to selection bias despite randomization being performed, such as differences in diastolic blood pressure between the two groups before the start of the trial. Furthermore, although the percentage change in FEV_1_ post-bronchodilator in the study group was significant, it was less than 10%, a threshold of minimal clinically important difference [43].

## Conclusions

Over a 12-week treatment period, *Rhodiola crenulata* did not improve the 6MWD but did improve tidal breathing and ventilation efficiency in patients with COPD during incremental maximum exercise, most likely due to improvements in workload. Further studies with a larger patient population are needed to confirm the effects of *Rhodiola crenulata* on the reduction of triceps skin thickness, protection against acute exacerbations of COPD, and improvements in CAT scores.

## Supporting Information

S1 File The protocol. The original protocol of the study.
(DOC)

S2 File List of abbreviations and symptom scaling system. The alphabetical list of abbreviations used in this study and symptom scaling system including oxygen-cost diagram, modified Medical Research Council dyspnea scale, BODE composite index, and baseline dyspnea index.
(DOC)

S3 File Permission to use the SGRQ. SGRQ: the St George’s Respiratory Questionnaire.
(PDF)

S4 File CONSORT 2010 Checklist (2). Checklist of information to report a randomized trial.
(DOC)
