# Supplementary material for: Adjunctive Treatment with Rhodiola Crenulata in Patients with Chronic Obstructive Pulmonary Disease – A Randomized Placebo Controlled Double Blind Clinical Trial
Source: PLoS One. 2015 Jun 22;10(6):e0128142. doi: 10.1371/journal.pone.0128142 (PMC4476627; doi:10.1371/journal.pone.0128142)
Supplement: S1 File — The original protocol of the study. (DOC) [file pone.0128142.s001.doc]

**PROTOCOL**  V1.1 12/04/2012

|  | | | | No：CSH-2012-C-023 | | |
| --- | --- | --- | --- | --- | --- | --- |
| Department | Department of Critical Care Medicine and Division of Pulmonary Medicine | | | | | |
| PI | Ming-Lung Chuang  phone：(04)24739595-34144 | | co-PI | | Yao-Chern Wang  phone：(04)24739595-34144 | |
| co-PI | James CC Wei  phone：(04)24739595-34714 | | co-PI | | Chi-Chang Yang  phone：(04)24730022-12415 | |
| co-PI | Ming-Chian Yu  phone：(04)24739595-34821 | | co-PI | | Tzu-Chin Wu  phone：(04)24739595-34725 | |
| co-PI | Thomas CY Tsao  phone：(04)24730022-11021 | | co-PI | | Shih-Pin Chen  phone：(04)24739595-34713 | |
| co-PI | Shiu-chong Liu  phone：(04)24739595-34720 | | co-PI | | Yao-Tong Wang  phone：(04)24739595-34723 | |
| Period | between 2012 Jan 1 and 2012 Dec 31 | | | | | |
| Protocol title | 紅景天輔助改善慢性阻塞性肺病之隨機雙盲安慰劑控制式臨床試驗及機轉 | | | | | |
|  | Rhodiola crenulata as an adjunctive therapy in patients with chronic obstructive pulmonary disease | | | | | |
|  | □否 | | | | | |
|  | | Granted from | | | | note |
| wage | | NT $74,952 | | | |  |
| materials | | NT $360,048 | | | |  |
| others | | NT $65,000 | | | |  |
| total | | NT $500,000 | | | |  |
| contact person | | Ming-Lung Chuang phone: 886-4-24739595 ext 34144  e-mail:cshy1292@csh.org.tw | | | | |

**Abstract**

| **（Keyword :** *COPD, Rhodiola L., clinical trial, biomarkers, spirometry, exercise testing, quality of life***）**  **BACKGROUNDS.** Chronic obstructive pulmonary disease (COPD) has been one of the top ten causes of death in our Taiwanese population. Most patients present dyspnea and exercise intolerance because of the deranged lungs and impaired skeletomuscular function. Recently, low-grade systemic inflammation plays a vital role in its pathogenesis. Despite the mainstay of treatment using the inhaled medications that may lead to slowdown the progression of impaired lung function, improve the quality of life, and reduce the episodes of acute exacerbation, in turn the systemic inflammation is on-going and is hardly controlled by local therapy.  Herb medicine like Rhodiola L extract capsule contains p-tryosol, salidroside, rosavin, pyridrde, rhodiosin and rhodionin and has its anti-inflammatory and anti-oxidative effects and minimum side-effect after a long-term use as reported in the literature.    **AIMS.** We hypothesize that add-on Rhodiola L extract capsule to the regimen of patients with moderate-to-severe COPD (1) may provide a potential of systemic effects of anti-inflammation and anti-oxidation for these patients, and these effects (2) may reflect in the improvement of patients’ physiological measurements, quality of life and exercise tolerance.  **METHODS**  [Study design] A randomized, double-blind, placebo-controlled clinical trial.  [Subjects] We will enroll 60 moderate-to-severe COPD patients aged 40-79 years, abstaining from cigarette smoking or maintaining a stable dose of cigarette consumption, no acute exacerbation of COPD, being stable for one month and longer, or not undergoing exercise training program. Patients should have no uncontrolled diabetes mellitus, uremia, chronic heart failure, cerebrovascular disease, uncontrolled anemia, late-stage malignant diseases, or other acute illness, or taking any systemic anti-inflammatory agents.  [Procedures and Randomisation] All elegible subjects with moderate-to-severe COPD will be enrolled after signing the informed consent. All subjects will undergo the first set of measurements as follows. All subjects will be allocated as the study designed. The study group will contain 40 subjects taking Rhodiola L extract capsule Rhodiola L 250 mg twice per day for 12 weeks and the control group will contain 20 subjects taking the placebo of the same dose for the same duration.  [Measurements Set] (1) demographics, (2) anthropometrics, (3) symptomatology: dyspnea index, cough, sputum, sleep, sex, (4) composite index: BODE, (5) quality of life, (6) lung function, 6MWT and exercise test, (7) COPD acute exacerbations, and (8) biomarkers: IL-1, TNF-α, CRP, MMP-9, IL-4, and IFN-γ.  [Primary endpoint] Difference of 6 minutes walking distance at week 12. |
| --- |

**V**

| **BACKGROUND**  In World COPD Day, held annually on the third Wednesday in November, GOLD National Leaders, often in concert with local physicians, nurses, and health care planners, have hosted many types of activities to raise awareness of COPD. It is estimated that one tenth of whole world people will be suffered from COPD and by 2020, COPD will be one of the top three causes of death^1^。  Chronic obstructive pulmonary disease (COPD) has been one of the top ten causes of death in our Taiwanese population^2^ and its incidence will increase in the future likewise that anticipated in the developed countries.[^1^](#_ENREF_1) Most patients present dyspnea and exercise intolerance because of the deranged lungs and/or heart and impaired skeletomuscular function. Recently, the low-grade systemic inflammation has been reported to play a pivotal role in its pathogenesis.^3,4^ Despite the mainstay of treatment using the inhaled medications (including long-acting bronchodilators plus corticosteroids)[^5^](#_ENREF_4) that lead to slowdown the progression of impaired lung function, to improve the quality of life (including psychosocial dimension), and to reduce the episodes of acute exacerbation, in turn the low-grade systemic inflammation is on-going. This process is hardly controlled by local therapy like inhaling medication to the lung.  Corticosteroids has been well-known by their systemic anti-inflammatory effect by oral administration; however, they are notorious by their systemic side-effects after a prolonged use.[^1^](#_ENREF_1) Roflumilast, a PDE4 inhibitor, presents anti-inflammation by inhibiting the intracellular c-AMP breakdown. It increases FEV_1_ when it is concurrently treated with seretide or tiotropium. However, it cannot go with theophylline for the patients.^6,7^ Moreover, it contains subtantial side effects such as nausea, vomiting, abdominal pain, diarrhea, sleep disturbance, and depression leading to average body weight loss of 2kg and withdrawal from the study. Antioxidant such as N-acetylcystein contains no effect on acute exacerbation.[^8^](#_ENREF_7) Infliximab, an anti-TNF-α, contains no benefit for COPD patients and potentially induces cancer and pneumonia.[^9^](#_ENREF_8)  Herbal medicines contain some benefits for patients with COPD regarding lung function and exercise capacity[^10^](#_ENREF_9); however, clinical outcomes are mixed due to different study design, pharmacological mechanisms, and different treatment duration and follow-up period.[^11^](#_ENREF_10) In addition, the measurements have been always focused on FEV_1_ which has been criticized to be the paramount variable for evaluation of this patient population. Rather, the quality of life and lung function variables other than FEV_1_ are probably more important than FEV_1_.  Rhodiola rosea (L.) is a precious phytomedicine that has grown in high altitudes and cold environments in Tibet and China for many centuries. In [Russia](http://en.wikipedia.org/wiki/Russia) and [Scandinavia](http://en.wikipedia.org/wiki/Scandinavia), Rhodiola rosea, also known as golden root, has been used for centuries to cope with the cold Siberian climate and stressful life. Recently, a C. elegans model of stress provided evidence of the adaptogenic effects of Rhodiola (Wiegant et al., 2009), and in rats, Rhodiola effectively prevented stress-induced changes in appetite, physical activity, weight gain, and estrus cycle (Darbinyan et al., 2007; Mattioli et al., 2009). Rhodiola rosea Rhodiola L extract capsule has its anti-inflammatory and anti-oxidative effects and protect muscle tissue during exercise (Abidov et al., 2004). It suppresses the increases of the enzyme activities of serum glutamic pyruvic transaminase, alkaline phosphatase, and creatine kinase elicited by noise (Zhu et al., 2004) so that it selectively attenuates stress-induced anorexia, providing functional anti-stress properties. However, studies on whether Rhodiola improves physical performance have been inconclusive, with some studies showing benefits (De Bock et al., 2004; Darbinyan et al., 2007; Li et al., 2008, Mattioli et al., 2009) and others reporting no significant effectiveness (Walker et al., 2007).  Animal tests have suggested a variety of beneficial effects of Rhodiola rosea extract (RRE) (Perfumi and Mattioli, 2007). Recently, the efficacy of RRE in the treatment of inflammatory conditions was revealed through rat models of carrageenan-induced paw oedema, formaldehyde-induced arthritis, and nystatin-induced paw oedema (Pooja et al., 2009). RRE was reported to exhibit inhibitory effects against acute and subacute inflammation at a dose of 250 mg/kg body weight. At 200 mg/kg daily, RRE also significantly decreased blood glucose and increased levels of reduced glutathione and the activities of glutathione reductase, glutathione S-transferase, glutathione peroxidase, catalase, and superoxide dismutase in the livers of mice (Kim et al., 2006). Moreover, a Rhodiola dietary supplement (30 mg/ml) supplied every other day was reported to significantly increase the lifespan of Drosophila melanogaster (Jafari et al., 2007).  Rhodiola has been demonstrated to function as an adaptogen due to its antioxidant potential (Chen etal., 2008). Adaptogens are distinct from other substances in their ability to balance endocrine hormones and the immune system and help the body maintain optimal homeostasis. Adaptogens are proposed to have a normalizing effect on the body and to be capable of either toning down the activity of hyperfunctioning systems or strengthening the activity of hypofunctioning systems.  Our previous results demonstrated no weight differences across treatment groups (P > 0.05). Furthermore, our pathological examination results (Chou Lin et al., 2011a) are consistent with previous reports (Kim et al., 2006; Pooja et al., 2009), suggesting that daily RRSS doses up to 200 mg/kg body weight and salidroside doses of 4.4 mg/kg body weight had no toxic effect on the brain, heart, liver, spleen, or kidneys in mice. The results of liver and kidney function tests also suggest that Rhodiola rosea extract is safe for use in vivo, at least over a 28-day period.  Among its active components, salidroside is the most widely investigated compound in Rhodiola rosea. Salidroside was reported to dose-dependently restore the H2O2-induced loss of mitochondrial membrane potential and elevate intracellular calcium levels (Zhang et al., 2007). Salidroside significantly reduced amyloid beta peptide (Abeta)-induced cytotoxicity in a dose-dependent manner and also reduced Abeta-mediated intracellular accumulation of reactive oxygen species and malondialdehyde, a product of lipid peroxides, by preventing Abeta-induced decline in antioxidant enzyme activity (Jang et al., 2003). These results suggest that salidroside has protective effects against oxidative stress-induced cell apoptosis (Zhang et al., 2007). Furthermore, salidroside has been reported to protect liver tissue from acetaminophen-induced oxidative damage via prevention or alleviation of intracellular glutathione depletion and oxidation. This finding suggests that salidroside may be a potential antidote against acetaminophen-induced hepatotoxicity (Wu et al., 2008).  More recently, salidroside has been suggested to possess protective effects against hypoxia-induced cardiomyocyte necrosis and apoptosis by increasing hypoxia-inducible factor-1 expression and subsequently upregulating vascular endothelial growth factor levels (Zhang et al., 2009). Another study suggests that salidroside possesses antiviral activity against coxsackievirus B3 and may represent a potential therapeutic agent for viral myocarditis (Wang et al., 2009). Based on the finding that salidroside from Rhodiola rosea significantly increased the mRNA expression of IL-10 and IFN-γ, but decreased tumour necrosis factor (TNF)-α and IL-2 mRNA expression, Wang et al. (2009) suggested that salidroside may prevent inflammatory responses and strengthen host resistance against virus infection by enhancing the expression of IL-10 and IFN-γ in heart tissues, and suppress myocardial apoptosis and myocardial dysfunction by inhibiting TNF-α and IL-2 mRNA expression. The above findings are partially inconsistent (IL-2) with the results of our study (Chou Lin et al., 2011a). We found that salidroside had immunomodulatory effects on both Th1 cytokines, i.e. IL-2 and IFN-γ, and Th2 cytokines, i.e. IL-4 and IL-10. This difference may be due to the different tissue (heart or serum), expression marker (mRNA or protein), and infection status (virus-infected or uninfected) examined. Furthermore, no dose-dependent immunomodulatory effect was observed for salidroside in our previous study (Chou Lin et al., 2011a). Interestingly, the cytokine modulation effects of salidroside were less prominent than those of RRSS, although the salidroside content in both groups was nearly equal. Our previous study showed that the effects of the individual components alone cannot substitute for or represent the cumulative effects of herbal medicine (Lin et al., 2006). However, this finding is quite different from our recent study, in which serum concentrations of IL-2, IL-4, and IL-10 but not IFN-gamma were significantly increased after treatment with 1 mg/kg body weight cinnamaldehyde, but not significantly affected by treatment with Cinnamomum osmophloeum leaf essential oil groups that contained similar cinnamaldehyde levels (Chou Lin et al., 2011b). Synergistic effects of salidroside and the class of rosavins have been demonstrated (Panossian et al., 2008), and these synergistic effects may underlie the less prominent cytokine modulation effects observed with salidroside compared to RRSS.  Our previous data suggest that increased secretion of both Th1- and Th2- pattern cytokines in mouse serum can be achieved with RRSS and salidroside treatment, and that the effect of RRSS on cytokine modulation in vivo is dependent on herb dosage and related to the treatment period. The effects of Rhodiola algida and Rhodiola imbricata extracts on the expression of Th1 and Th2 cytokines have recently been analyzed in human peripheral lymphocytes (Li et al., 2009; Mishra et al., 2009). Although different species of the Rhodiola genus were tested, our results are consistent with those of Rhodiola algida, suggesting that both Th1 and Th2 cytokines were upregulated in vivo when mice were fed RRSS. However, our in vivo data differ from those of Rhodiola imbricate, which led to increased expression levels of Th1 cytokines including IL-2 and TNF-α but not Th2 cytokines (IL-4 and IL-10) in vitro (Chou Lin et al., 2011a). Moreover, our results demonstrated that IL-2 and IL-4 quickly respond to RRSS treatment in a similar manner, and that IL-10 and IFN-γ increase to higher levels than IL-2 and IL-4 (Chou Lin et al., 2011a). The higher concentrations of IL-2 and IL-4 produced by RRSS treatment may also reflect the balance between Th1 and Th2 cytokines. Furthermore, the strongest cytokine modulation effects were observed in mice treated with the highest RRSS dosage and synergistic effects may account for the more prominent cytokine modulation effects of RRSS compared to salidroside alone. In addition, no side effects or toxicity were observed in the mice administered RRSS, with no significant weight differences, pathological changes, or abnormal biochemistry indices observed across all groups (Chou Lin et al., 2011a).  **STUDY AIMS**  We hypothesize that add-on Rhodiola L extract capsule to the regular regimen for patients with moderate-to-severe COPD who have a history of stable medication regimen (1) may provide a potential of systemic anti-inflammation and anti-oxidation for these patients, and (2) these effects may reflect in the improvement of the patients’ symptomatology, physiological measurements, quality of life and exercise tolerance.  **REFERENCES**   1. Top ten causes of death in Taiwan. <http://www.doh.gov.tw/CHT2006/DM/DM2_2.aspx?now_fod_list_no=11962&class_no=440&level_no=4>. 2010 2. GOLD C. Global Strategy for the Diagnosis, Management, and Prevention of Chronic Obstructive Pulmonary Disease (UPDATED 2010). Disclosure forms for GOLD Committees are posted on the GOLD Website, [www.goldcopd.org](http://www.goldcopd.org/) 2010 3. Reid WD, Rurak J, Harris RL. Skeletal muscle response to inflammation--lessons for chronic obstructive pulmonary disease. Crit Care Med 2009; 37:S372-383 4. Remels AH, Gosker HR, van der Velden J, et al. Systemic inflammation and skeletal muscle dysfunction in chronic obstructive pulmonary disease: state of the art and novel insights in regulation of muscle plasticity. Clin Chest Med 2007; 28:537-552, vi 5. Calverley PM, Anderson JA, Celli B, et al. Salmeterol and fluticasone propionate and survival in chronic obstructive pulmonary disease. N Engl J Med 2007; 356:775-789 6. Calverley PM, Rabe KF, Goehring UM, et al. Roflumilast in symptomatic chronic obstructive pulmonary disease: two randomised clinical trials. Lancet 2009; 374:685-694 7. Fabbri LM, Calverley PM, Izquierdo-Alonso JL, et al. Roflumilast in moderate-to-severe chronic obstructive pulmonary disease treated with longacting bronchodilators: two randomised clinical trials. Lancet 2009; 374:695-703 8. Decramer M, Rutten-van Molken M, Dekhuijzen PN, et al. Effects of N-acetylcysteine on outcomes in chronic obstructive pulmonary disease (Bronchitis Randomized on NAC Cost-Utility Study, BRONCUS): a randomised placebo-controlled trial. Lancet 2005; 365:1552-1560 9. Rennard SI, Fogarty C, Kelsen S, et al. The safety and efficacy of infliximab in moderate to severe chronic obstructive pulmonary disease. Am J Respir Crit Care Med 2007; 175:926-934 10. Gross D, Shenkman Z, Bleiberg B, et al. Ginseng improves pulmonary functions and exercise capacity in patients with COPD. Monaldi Arch Chest Dis 2002; 57:242-246 11. Guo R, Pittler MH, Ernst E. Herbal medicines for the treatment of COPD: a systematic review. Eur Respir J 2006; 28:330-338 12. Borg G. Psychophysical bases of perceived exertion. Med Sci Sports Exercise 1982; 14:377-381 13. Brooks S. Surveillance for respiratory hazards. ATS News 1982; 8:12–16 14. Chuang ML, Lee CH, Lin IF. Using the oxygen-cost diagram in ramp-slope selection for dyspneic patients. Intern Med 2010; 49:1325-1332 15. Celli BR, Cote CG, Marin JM, et al. The body-mass index, airflow obstruction, dyspnea, and exercise capacity index in chronic obstructive pulmonary disease. N Engl J Med 2004; 350:1005-1012 16. Guyatt G. Measuring health status in chronic airflow limitation. Eur Respir J 1988; 1:560-564 17. Jones PW. Health status measurement in chronic obstructive pulmonary disease. Thorax 2001; 56:880-887 18. Zigmond AS, Snaith RP. The hospital anxiety and depression scale. Acta Psychiatr Scand 1983; 67:361-370 19. Chuang ML, Lin IF, Wasserman K. The body weight-walking distance product as related to lung function, anaerobic threshold and peak VO2 in COPD patients. Respir Med 2001; 95:618-626 20. ATS/ACCP Statement on cardiopulmonary exercise testing. Am J Respir Crit Care Med 2003; 167:211-277 21. Jenkins S, Hill K, Cecins NM. State of the art: how to set up a pulmonary rehabilitation program. Respirology; 15:1157-1173 22. Burrows B, Kasik JE, Niden AH, et al. Clinical usefulness of the single breath pulmonary diffusing capacity test. Am Rev Respir Dis 1961; 84:789-806. 23. Goldman HI BM. Respiratory function tests - Normal values at median altitudes and the prediction of normal results. Am Rev Respir Dis 1959; 79:457-467 24. Knudson RJ, Slatin RC, Lebowitz MD, et al. The maximal expiratory flow-volume curve. Normal standards, variability, and effects of age. Am Rev Respir Dis 1976; 113:587-600 25. Wasserman K, Hansen JE, Sue DY, et al. Clinical exercise testing. In: Wasserman K, ed. Principles of exercise testing and interpretation. Philadelphia: Lippicott Williams & Wilkins, 2005; 133-159. 26. Guyatt GH, Pugsley SO, Sullivan MJ, et al. Effect of encouragement on walking test performance. Thorax 1984; 39:818-822 27. Sue DY, Wasserman K, Moricca RB, et al. Metabolic acidosis during exercise in patients with chronic obstructive pulmonary disease. Use of the V-slope method for anaerobic threshold determination. Chest 1988; 94:931-93 28. Wasserman K, Hansen JE, Sue DY, et al. Normal values. In: Wasserman K, ed. Principles of exercise testing and interpretation. Philadelphia: Lippincot Williams & Wilkins, 2005; 160-182. 29. [Abidov M](http://www.ncbi.nlm.nih.gov/sites/entrez?Db=pubmed&Cmd=Search&Term="Abidov M"%5BAuthor%5D&itool=EntrezSystem2.PEntrez.Pubmed.Pubmed_ResultsPanel.Pubmed_DiscoveryPanel.Pubmed_RVAbstractPlus), [Grachev S](http://www.ncbi.nlm.nih.gov/sites/entrez?Db=pubmed&Cmd=Search&Term="Grachev S"%5BAuthor%5D&itool=EntrezSystem2.PEntrez.Pubmed.Pubmed_ResultsPanel.Pubmed_DiscoveryPanel.Pubmed_RVAbstractPlus), [Seifulla RD](http://www.ncbi.nlm.nih.gov/sites/entrez?Db=pubmed&Cmd=Search&Term="Seifulla RD"%5BAuthor%5D&itool=EntrezSystem2.PEntrez.Pubmed.Pubmed_ResultsPanel.Pubmed_DiscoveryPanel.Pubmed_RVAbstractPlus), [Ziegenfuss TN](http://www.ncbi.nlm.nih.gov/sites/entrez?Db=pubmed&Cmd=Search&Term="Ziegenfuss TN"%5BAuthor%5D&itool=EntrezSystem2.PEntrez.Pubmed.Pubmed_ResultsPanel.Pubmed_DiscoveryPanel.Pubmed_RVAbstractPlus). 2004. Extract of *Rhodiola rosea* radix reduces the level of C-reactive protein and creatinine kinase in the blood. *Bull Exp Biol Med* **138**; 63-64 30. Chen TS, Liou SY, Chang YL. 2008. [Antioxidant evaluation of three adaptogen extracts.](http://www.ncbi.nlm.nih.gov/pubmed/19051347?ordinalpos=18&itool=Email.EmailReport.Pubmed_ReportSelector.Pubmed_RVDocSum) *Am J Chin Med* **36**; 1209-1217. 31. Chou Lin SS, Chin LW, Chao PC, Lai YY, Lin LY, Chou MY, Chou MC, Wei JCC, Yang CC. *In vivo* Th1 and Th2 cytokine modulation effects of *Rhodiola rosea*-soaked solution and its major constituent, salidroside. Phytotherapy Research 2011a, DOI: 10.1002/ptr.3451, in press. 32. Chou Lin SS, Lu TM, Chao PC, Lai YY, Tsai HT, Chen CS, Lee YP, Chen SJ, Chou MC, Yang CC**.** 2011. *In vivo* cytokine modulation effects of *cinnamaldehyde,* the major constituent of leaf essential oil from *Cinnamomum osmophloeum* Kaneh. Phytotherapy Research 2011b, DOI: 10.1002/ptr.3419, in press. 33. Darbinyan V, Aslanyan G, Amroyan E, Gabrielyan E, Malmström C, Panossian A. 2007. Clinical trial of *Rhodiola rosea* L. extract SHR-5 in the treatment of mild to moderate depression. *Nord J Psychiatry* **61**; 343-348. 34. De Bock K, Eijnde BO, Ramaekers M, Hespel P. 2004. Acute Rhodiola rosea intake can improve endurance exercise performance. *Int J Sport Nutr Exerc Metabol* **14**; 298-307. 35. Ganzera M, Yayla Y, Khan IA. 2001. Analysis of the marker compounds of *Rhodiola rosea* L. (Golden Root) by reversed phase high performance liquid chromatography. *Chem Pharm Bull* **49(4)**; 465-467. 36. Ho CC, Lin SS, Chou MY, Chen FL, Hu CC, Chen CS, Lu GY, Yang CC. 2005. Effects of CAPE-like compounds on HIV replication *in vitro* and modulation of cytokines *in vivo*. *J Antimicrob Chemother* **56**; 372-379. 37. Hsieh CK, Lin CF, Ni CL, Chien MY, Chang JM, Hsieh CC, Chen CC. 2006. Quantitative analysis of salidroside and p-tyrosol in rhizoma Rhodiola. *J Chin Med* **17**; 151-158. 38. Huang TH, Yang CC, Ding SJ, Yen M, Kao CT, Chou MY. 2005. Inflammatory cytokines reaction elicited by root-end filling materials. *J Biomed Mater Res* **72B**; 123-128. 39. [Jafari M](http://www.ncbi.nlm.nih.gov/sites/entrez?Db=pubmed&Cmd=Search&Term="Jafari M"%5BAuthor%5D&itool=EntrezSystem2.PEntrez.Pubmed.Pubmed_ResultsPanel.Pubmed_DiscoveryPanel.Pubmed_RVAbstractPlus), [Felgner JS](http://www.ncbi.nlm.nih.gov/sites/entrez?Db=pubmed&Cmd=Search&Term="Felgner JS"%5BAuthor%5D&itool=EntrezSystem2.PEntrez.Pubmed.Pubmed_ResultsPanel.Pubmed_DiscoveryPanel.Pubmed_RVAbstractPlus), [Bussel II](http://www.ncbi.nlm.nih.gov/sites/entrez?Db=pubmed&Cmd=Search&Term="Bussel II"%5BAuthor%5D&itool=EntrezSystem2.PEntrez.Pubmed.Pubmed_ResultsPanel.Pubmed_DiscoveryPanel.Pubmed_RVAbstractPlus) [Hutchili T](http://www.ncbi.nlm.nih.gov/sites/entrez?Db=pubmed&Cmd=Search&Term="Hutchili T"%5BAuthor%5D&itool=EntrezSystem2.PEntrez.Pubmed.Pubmed_ResultsPanel.Pubmed_DiscoveryPanel.Pubmed_RVAbstractPlus), [Khodayari B](http://www.ncbi.nlm.nih.gov/sites/entrez?Db=pubmed&Cmd=Search&Term="Khodayari B"%5BAuthor%5D&itool=EntrezSystem2.PEntrez.Pubmed.Pubmed_ResultsPanel.Pubmed_DiscoveryPanel.Pubmed_RVAbstractPlus), [Rose MR](http://www.ncbi.nlm.nih.gov/sites/entrez?Db=pubmed&Cmd=Search&Term="Rose MR"%5BAuthor%5D&itool=EntrezSystem2.PEntrez.Pubmed.Pubmed_ResultsPanel.Pubmed_DiscoveryPanel.Pubmed_RVAbstractPlus), [Vince-Cruz C](http://www.ncbi.nlm.nih.gov/sites/entrez?Db=pubmed&Cmd=Search&Term="Vince-Cruz C"%5BAuthor%5D&itool=EntrezSystem2.PEntrez.Pubmed.Pubmed_ResultsPanel.Pubmed_DiscoveryPanel.Pubmed_RVAbstractPlus), [Mueller LD](http://www.ncbi.nlm.nih.gov/sites/entrez?Db=pubmed&Cmd=Search&Term="Mueller LD"%5BAuthor%5D&itool=EntrezSystem2.PEntrez.Pubmed.Pubmed_ResultsPanel.Pubmed_DiscoveryPanel.Pubmed_RVAbstractPlus). 2007. [Rhodiola: a promising anti-aging Chinese herb.](http://www.ncbi.nlm.nih.gov/pubmed/17990971?ordinalpos=59&itool=Email.EmailReport.Pubmed_ReportSelector.Pubmed_RVDocSum) *Rejuvenation Res* **10**; 587-602. 40. [Jang SI](http://www.ncbi.nlm.nih.gov/sites/entrez?Db=pubmed&Cmd=Search&Term="Jang SI"%5BAuthor%5D&itool=EntrezSystem2.PEntrez.Pubmed.Pubmed_ResultsPanel.Pubmed_DiscoveryPanel.Pubmed_RVAbstractPlus), [Pae HO](http://www.ncbi.nlm.nih.gov/sites/entrez?Db=pubmed&Cmd=Search&Term="Pae HO"%5BAuthor%5D&itool=EntrezSystem2.PEntrez.Pubmed.Pubmed_ResultsPanel.Pubmed_DiscoveryPanel.Pubmed_RVAbstractPlus), [Choi BM](http://www.ncbi.nlm.nih.gov/sites/entrez?Db=pubmed&Cmd=Search&Term="Choi BM"%5BAuthor%5D&itool=EntrezSystem2.PEntrez.Pubmed.Pubmed_ResultsPanel.Pubmed_DiscoveryPanel.Pubmed_RVAbstractPlus), [Oh GS](http://www.ncbi.nlm.nih.gov/sites/entrez?Db=pubmed&Cmd=Search&Term="Oh GS"%5BAuthor%5D&itool=EntrezSystem2.PEntrez.Pubmed.Pubmed_ResultsPanel.Pubmed_DiscoveryPanel.Pubmed_RVAbstractPlus), [Jeong S](http://www.ncbi.nlm.nih.gov/sites/entrez?Db=pubmed&Cmd=Search&Term="Jeong S"%5BAuthor%5D&itool=EntrezSystem2.PEntrez.Pubmed.Pubmed_ResultsPanel.Pubmed_DiscoveryPanel.Pubmed_RVAbstractPlus), [Lee HJ](http://www.ncbi.nlm.nih.gov/sites/entrez?Db=pubmed&Cmd=Search&Term="Lee HJ"%5BAuthor%5D&itool=EntrezSystem2.PEntrez.Pubmed.Pubmed_ResultsPanel.Pubmed_DiscoveryPanel.Pubmed_RVAbstractPlus), [Kim HY](http://www.ncbi.nlm.nih.gov/sites/entrez?Db=pubmed&Cmd=Search&Term="Kim HY"%5BAuthor%5D&itool=EntrezSystem2.PEntrez.Pubmed.Pubmed_ResultsPanel.Pubmed_DiscoveryPanel.Pubmed_RVAbstractPlus), [Kang KJ](http://www.ncbi.nlm.nih.gov/sites/entrez?Db=pubmed&Cmd=Search&Term="Kang KJ"%5BAuthor%5D&itool=EntrezSystem2.PEntrez.Pubmed.Pubmed_ResultsPanel.Pubmed_DiscoveryPanel.Pubmed_RVAbstractPlus), [Yun YG](http://www.ncbi.nlm.nih.gov/sites/entrez?Db=pubmed&Cmd=Search&Term="Yun YG"%5BAuthor%5D&itool=EntrezSystem2.PEntrez.Pubmed.Pubmed_ResultsPanel.Pubmed_DiscoveryPanel.Pubmed_RVAbstractPlus), [Kim YC](http://www.ncbi.nlm.nih.gov/sites/entrez?Db=pubmed&Cmd=Search&Term="Kim YC"%5BAuthor%5D&itool=EntrezSystem2.PEntrez.Pubmed.Pubmed_ResultsPanel.Pubmed_DiscoveryPanel.Pubmed_RVAbstractPlus), [Chung HT](http://www.ncbi.nlm.nih.gov/sites/entrez?Db=pubmed&Cmd=Search&Term="Chung HT"%5BAuthor%5D&itool=EntrezSystem2.PEntrez.Pubmed.Pubmed_ResultsPanel.Pubmed_DiscoveryPanel.Pubmed_RVAbstractPlus). 2003. Salidroside from *Rhodiola sachalinensis* protects neuronal PC12 cells against cytotoxicity induced by amyloid-beta. [*Immunopharmacol Immunotoxicol*](javascript:AL_get(this, 'jour', 'Immunopharmacol Immunotoxicol.');) **25**; 295-304. 41. Kim SH, Hyun SH, Choung SY. 2006. Antioxidative effects of *Cinnamomi cassiae* and *Rhodiola rosea* extracts in liver of diabetic mice. [*Biofactors*](javascript:AL_get(this, 'jour', 'Biofactors.');) **26**; 209-219. 42. Kucinskaite A, Briedis V, Savickas A. 2004. Experimental analysis of therapeutic properties of *Rhodiola rosea* L. and its possible application in medicine. (Lithuanian) *Medicina* (Kaunas). 40; 614-619. 43. Li HB, Ge YK, Zheng XX, Zhang L. 2008. [Salidroside stimulated glucose uptake in skeletal muscle cells by activating AMP-activated protein kinase.](http://www.ncbi.nlm.nih.gov/pubmed/18501890?ordinalpos=38&itool=Email.EmailReport.Pubmed_ReportSelector.Pubmed_RVDocSum) *Eur J Pharmacol* **588**; 165-169. 44. Li HX, Sze SCW, Tong Y, Ng TB. 2009. Production of Th1- and Th2-dependent cytokines induced by the Chinese medicine herb, *Rhodiola algida*, on human peripheral blood monocytes. [*J Ethnopharmacol*](http://www.sciencedirect.com/science/journal/03788741) **123**; 257-266. 45. Lin SJ, Chen CS, Ho CC, Lin SS, Shih HT, Lee IP, Chou MY, Kao CT, Chen FL, Ho YC, Hsieh KH, Huang CR, Yang CC. 2006. *In vitro* anti-microbial and *in vivo* cytokines modulation effects of different prepared Chinese herbal medicines. *Food Chem Toxicol* **44**; 2078-2085. 46. Lin SJ, Tsai JH, Tsai CH, Hsu HT, Lin YC, Xu FL, Yang CC. 2004. The *in vivo* effects of cytokines modulation for balb/c mice fed with a combined Chinese herb-soaked solution, Yifei-Ruennhou tea. *Immunopharacol Immunotoxicol* **26**; 435-444. 47. Lin SJ, Tsai JH, Tsai CH, Lin YC, Hsu HT, Xu FL, Yang CC. 2005a. The *in vivo* effects of cytokine modulation for Balb/C mice given *Canavalia ensiformis* (L.) seeds with different heat treatment. *Food Chem* **91**; 139-145. 48. Lin SS, Chou MY, Ho CC, Kao CT, Tsai CH, Wang L, Yang CC. 2005b. Study of the viral infections and cytokines associated with recurrent aphthous ulceration. *Microb Infect* **7**; 635-644. 49. MacNeil IA, Suda T, Moore KW, Mosmann TR, Zlotnik A. 1990. IL-10, a novel growth cofactor for mature and immature T cells. *J Immunol* **145**; 4167-4173. 50. Mao Y, Li Y, Yao N. 2007. Simultaneous determination of salidroside and tyrosol in extracts of Rhodiola L. by microwave assisted extraction and high-performance liquid chromatography. *J Pharmaceut Biomed Anal* **45**; 510-515. 51. Marcelletti JF, Katz DH. 1991. Elicitation of antigen-induced primary and secondary murine IgE antibody responses *in vitro*. *Cell Immunol* **135**; 471-489. 52. [Mattioli L, Funari C, Perfumi M. 2009. Effects of *Rhodiola rosea* L. extract on behavioural and physiological alterations induced by chronic mild stress in female rats. *J Psychopharmacol* **23**; 130-142](http://www.ncbi.nlm.nih.gov/pubmed/18515456). 53. [Mishra KP](http://www.ncbi.nlm.nih.gov/pubmed?term="Mishra KP"%5BAuthor%5D&itool=EntrezSystem2.PEntrez.Pubmed.Pubmed_ResultsPanel.Pubmed_RVAbstract), [Ganju L](http://www.ncbi.nlm.nih.gov/pubmed?term="Ganju L"%5BAuthor%5D&itool=EntrezSystem2.PEntrez.Pubmed.Pubmed_ResultsPanel.Pubmed_RVAbstract), [Chanda S](http://www.ncbi.nlm.nih.gov/pubmed?term="Chanda S"%5BAuthor%5D&itool=EntrezSystem2.PEntrez.Pubmed.Pubmed_ResultsPanel.Pubmed_RVAbstract), [Karan D](http://www.ncbi.nlm.nih.gov/pubmed?term="Karan D"%5BAuthor%5D&itool=EntrezSystem2.PEntrez.Pubmed.Pubmed_ResultsPanel.Pubmed_RVAbstract), [Sawhney RC](http://www.ncbi.nlm.nih.gov/pubmed?term="Sawhney RC"%5BAuthor%5D&itool=EntrezSystem2.PEntrez.Pubmed.Pubmed_ResultsPanel.Pubmed_RVAbstract). 2009. [Aqueous extract of *Rhodiola imbricata* rhizome stimulates Toll-like receptor 4, granzyme-B and Th1 cytokines *in vitro*](http://www.sciencedirect.com/science?_ob=ArticleURL&_udi=B7GW1-4SSGCDM-3&_user=1565457&_coverDate=01%2F31%2F2009&_rdoc=5&_fmt=high&_orig=browse&_srch=doc-info(%23toc%2320445%232009%23997859998%23855049%23FLA%23display%23Volume)&_cdi=20445&_sort=d&_docanchor=&_ct=10&_acct=C000053757&_version=1&_urlVersion=0&_userid=1565457&md5=9d3aa7b6a974493e6996ba13f6d2f889). *Immunobiology* **214**: 27-31. 54. Panossian A, Nikoyan N, Ohanyan N, Hovhannisyan A, Abrahamyan H, Gabrielyan E, Wikman G. 2008. Comparative study of Rhodiola preparations on behavioral despair of rats. *Phytomedicine* **15**; 84-91. 55. Perfumi M, Mattioli L. 2007. Adaptogenic and central nervous system effects of single doses of 3% rosavin and 1% salidroside *Rhodiola rosea* L. extract in mice. *Phytother Res* **21**; 37-43. 56. [Pooja](http://www.ncbi.nlm.nih.gov/sites/entrez?Db=pubmed&Cmd=Search&Term="Pooja"%5BCorporate Author%5D&itool=EntrezSystem2.PEntrez.Pubmed.Pubmed_ResultsPanel.Pubmed_DiscoveryPanel.Pubmed_RVAbstractPlus) Bawa AS, [Khanum F](http://www.ncbi.nlm.nih.gov/sites/entrez?Db=pubmed&Cmd=Search&Term="Khanum F"%5BAuthor%5D&itool=EntrezSystem2.PEntrez.Pubmed.Pubmed_ResultsPanel.Pubmed_DiscoveryPanel.Pubmed_RVAbstractPlus). 2009. Anti-inflammatory activity of *Rhodiola rosea*-"a second-generation adaptogen". *Phytother Res* **23**; 1099-1102. 57. Seder RA, Paul WE. 1994. Acquisition of lymphokine-producing phenotype by CD4+ T cells. *Annu Rev Immunol* **12**; 635-673. 58. [Tolonen A](http://www.ncbi.nlm.nih.gov/sites/entrez?Db=pubmed&Cmd=Search&Term="Tolonen A"%5BAuthor%5D&itool=EntrezSystem2.PEntrez.Pubmed.Pubmed_ResultsPanel.Pubmed_DiscoveryPanel.Pubmed_RVAbstractPlus), [Pakonen M](http://www.ncbi.nlm.nih.gov/sites/entrez?Db=pubmed&Cmd=Search&Term="Pakonen M"%5BAuthor%5D&itool=EntrezSystem2.PEntrez.Pubmed.Pubmed_ResultsPanel.Pubmed_DiscoveryPanel.Pubmed_RVAbstractPlus), [Hohtola A](http://www.ncbi.nlm.nih.gov/sites/entrez?Db=pubmed&Cmd=Search&Term="Hohtola A"%5BAuthor%5D&itool=EntrezSystem2.PEntrez.Pubmed.Pubmed_ResultsPanel.Pubmed_DiscoveryPanel.Pubmed_RVAbstractPlus), [Jalonen J](http://www.ncbi.nlm.nih.gov/sites/entrez?Db=pubmed&Cmd=Search&Term="Jalonen J"%5BAuthor%5D&itool=EntrezSystem2.PEntrez.Pubmed.Pubmed_ResultsPanel.Pubmed_DiscoveryPanel.Pubmed_RVAbstractPlus). 2003. Phenylpropanoid glycosides from *Rhodiola rosea*. [*Chem. Pharmaceut. Bull.*](javascript:AL_get(this, 'jour', 'Chem Pharm Bull (Tokyo).');) **51**; 467-470. 59. Tsai CH, Tsai HH, Yang YY, Ku CS, Hu CC, Lin HW, Yang CC. 2000. The *in vivo* effects of cytokines modulation in Balb/C mice fed with *Canavalia ensiformis* seeds. *J Biomed Lab Sci* **12**; 31-34. 60. Walker TB, Altobelli SA, Caprihan A, Robergs RA. 2007. Failure of *Rhodiola rosea* to alter skeletal muscle phosphate kinetics in trained men. *Metabolism* **56**; 1111-1117. 61. [Wang H](http://www.ncbi.nlm.nih.gov/sites/entrez?Db=pubmed&Cmd=Search&Term="Wang H"%5BAuthor%5D&itool=EntrezSystem2.PEntrez.Pubmed.Pubmed_ResultsPanel.Pubmed_DiscoveryPanel.Pubmed_RVAbstractPlus), [Ding Y](http://www.ncbi.nlm.nih.gov/sites/entrez?Db=pubmed&Cmd=Search&Term="Ding Y"%5BAuthor%5D&itool=EntrezSystem2.PEntrez.Pubmed.Pubmed_ResultsPanel.Pubmed_DiscoveryPanel.Pubmed_RVAbstractPlus), [Zhou J](http://www.ncbi.nlm.nih.gov/sites/entrez?Db=pubmed&Cmd=Search&Term="Zhou J"%5BAuthor%5D&itool=EntrezSystem2.PEntrez.Pubmed.Pubmed_ResultsPanel.Pubmed_DiscoveryPanel.Pubmed_RVAbstractPlus), [Sun X](http://www.ncbi.nlm.nih.gov/sites/entrez?Db=pubmed&Cmd=Search&Term="Sun X"%5BAuthor%5D&itool=EntrezSystem2.PEntrez.Pubmed.Pubmed_ResultsPanel.Pubmed_DiscoveryPanel.Pubmed_RVAbstractPlus), [Wang S](http://www.ncbi.nlm.nih.gov/sites/entrez?Db=pubmed&Cmd=Search&Term="Wang S"%5BAuthor%5D&itool=EntrezSystem2.PEntrez.Pubmed.Pubmed_ResultsPanel.Pubmed_DiscoveryPanel.Pubmed_RVAbstractPlus). 2009. The *in vitro* and *in vivo* antiviral effects of salidroside from *Rhodiola rosea* L. against coxsackievirus B3. [*Phytomedicine*](javascript:AL_get(this, 'jour', 'Phytomedicine.');) **16**; 146-155. 62. Wiegant FAC, Surinova S, Ytsma E, Langelaar-Makkinje M, Wikman G, Post JA. 2009. Plant adaptogens increase lifespan and stress resistance in *C. elegans*. *Biogerontology* **10**; 27-42. 63. Wu YL, Piao DM, Han XH, Nan JX. 2008. Protective effects of salidroside against acetaminophen-induced toxicity in mice. *Biol Pharmaceut Bull* **31**; 1523-1529. 64. [Zhang J](http://www.ncbi.nlm.nih.gov/sites/entrez?Db=pubmed&Cmd=Search&Term="Zhang J"%5BAuthor%5D&itool=EntrezSystem2.PEntrez.Pubmed.Pubmed_ResultsPanel.Pubmed_DiscoveryPanel.Pubmed_RVAbstractPlus), [Liu A](http://www.ncbi.nlm.nih.gov/sites/entrez?Db=pubmed&Cmd=Search&Term="Liu A"%5BAuthor%5D&itool=EntrezSystem2.PEntrez.Pubmed.Pubmed_ResultsPanel.Pubmed_DiscoveryPanel.Pubmed_RVAbstractPlus), [Hou R](http://www.ncbi.nlm.nih.gov/sites/entrez?Db=pubmed&Cmd=Search&Term="Hou R"%5BAuthor%5D&itool=EntrezSystem2.PEntrez.Pubmed.Pubmed_ResultsPanel.Pubmed_DiscoveryPanel.Pubmed_RVAbstractPlus), [Zhang J](http://www.ncbi.nlm.nih.gov/sites/entrez?Db=pubmed&Cmd=Search&Term="Zhang J"%5BAuthor%5D&itool=EntrezSystem2.PEntrez.Pubmed.Pubmed_ResultsPanel.Pubmed_DiscoveryPanel.Pubmed_RVAbstractPlus), [Jia X](http://www.ncbi.nlm.nih.gov/sites/entrez?Db=pubmed&Cmd=Search&Term="Jia X"%5BAuthor%5D&itool=EntrezSystem2.PEntrez.Pubmed.Pubmed_ResultsPanel.Pubmed_DiscoveryPanel.Pubmed_RVAbstractPlus), [Jiang W](http://www.ncbi.nlm.nih.gov/sites/entrez?Db=pubmed&Cmd=Search&Term="Jiang W"%5BAuthor%5D&itool=EntrezSystem2.PEntrez.Pubmed.Pubmed_ResultsPanel.Pubmed_DiscoveryPanel.Pubmed_RVAbstractPlus), [Chen J](http://www.ncbi.nlm.nih.gov/sites/entrez?Db=pubmed&Cmd=Search&Term="Chen J"%5BAuthor%5D&itool=EntrezSystem2.PEntrez.Pubmed.Pubmed_ResultsPanel.Pubmed_DiscoveryPanel.Pubmed_RVAbstractPlus). 2009. [Salidroside protects cardiomyocyte against hypoxia-induced death: a HIF-1alpha-activated and VEGF-mediated pathway.](http://www.ncbi.nlm.nih.gov/pubmed/19326475?ordinalpos=4&itool=Email.EmailReport.Pubmed_ReportSelector.Pubmed_RVDocSum) *Eur J Pharmacol* **607**; 6-14 65. Zhang L, Yu H, Sun Y, Lin X, Chen B, Tan C, Cao G, Wang Z. 2007. [Protective effects of salidroside on hydrogen peroxide-induced apoptosis in SH-SY5Y human neuroblastoma cells.](http://www.ncbi.nlm.nih.gov/pubmed/17349619?ordinalpos=78&itool=Email.EmailReport.Pubmed_ReportSelector.Pubmed_RVDocSum) *Eur J Pharmacol* **564**; 18-25. 66. [Zhu BW](http://www.ncbi.nlm.nih.gov/sites/entrez?Db=pubmed&Cmd=Search&Term="Zhu BW"%5BAuthor%5D&itool=EntrezSystem2.PEntrez.Pubmed.Pubmed_ResultsPanel.Pubmed_DiscoveryPanel.Pubmed_RVAbstractPlus), [Sun YM](http://www.ncbi.nlm.nih.gov/sites/entrez?Db=pubmed&Cmd=Search&Term="Sun YM"%5BAuthor%5D&itool=EntrezSystem2.PEntrez.Pubmed.Pubmed_ResultsPanel.Pubmed_DiscoveryPanel.Pubmed_RVAbstractPlus), [Yun X](http://www.ncbi.nlm.nih.gov/sites/entrez?Db=pubmed&Cmd=Search&Term="Yun X"%5BAuthor%5D&itool=EntrezSystem2.PEntrez.Pubmed.Pubmed_ResultsPanel.Pubmed_DiscoveryPanel.Pubmed_RVAbstractPlus), [Han S](http://www.ncbi.nlm.nih.gov/sites/entrez?Db=pubmed&Cmd=Search&Term="Han S"%5BAuthor%5D&itool=EntrezSystem2.PEntrez.Pubmed.Pubmed_ResultsPanel.Pubmed_DiscoveryPanel.Pubmed_RVAbstractPlus), [Piao ML](http://www.ncbi.nlm.nih.gov/sites/entrez?Db=pubmed&Cmd=Search&Term="Piao ML"%5BAuthor%5D&itool=EntrezSystem2.PEntrez.Pubmed.Pubmed_ResultsPanel.Pubmed_DiscoveryPanel.Pubmed_RVAbstractPlus), [Murata Y](http://www.ncbi.nlm.nih.gov/sites/entrez?Db=pubmed&Cmd=Search&Term="Murata Y"%5BAuthor%5D&itool=EntrezSystem2.PEntrez.Pubmed.Pubmed_ResultsPanel.Pubmed_DiscoveryPanel.Pubmed_RVAbstractPlus), [Tada M](http://www.ncbi.nlm.nih.gov/sites/entrez?Db=pubmed&Cmd=Search&Term="Tada M"%5BAuthor%5D&itool=EntrezSystem2.PEntrez.Pubmed.Pubmed_ResultsPanel.Pubmed_DiscoveryPanel.Pubmed_RVAbstractPlus). 2004. Resistance imparted by traditional Chinese medicines to the acute change of glutamic pyruvic transaminase, alkaline phosphatase and creatine kinase activities in rat blood caused by noise. *Biosci Biotechnol Biochem* **68**; 1160-1163. 67. Zigmond AS, Snaith RP. The Hospital Anxiety and Depression Scale. Acta Psychiatr. Scand. 1983; 67: 361-370. |
| --- |

| **METHODS**  [Study design] A randomized, double-blind, placebo-controlled parallel clinical trial.  Randomization,  n=60  COPD  Study group, n=40  Week 0  Week 4  Week 8  Week 12  Control group, n=20  [Inclusion criteria] We will enroll 60 subjects with:   1. moderate-to-severe COPD patients 2. aged 40-79 years, 3. abstaining from cigarette smoking or maintaining a stable dose of cigarette consumption, 4. no acute exacerbation of COPD, 5. clinically being stable for one month and longer, 6. not undergoing exercise training program.   [Exclusion criteria] Patients should have no   1. uncontrolled diabetes mellitus by plasma fasting sugar >200 mg/dl, 2. uremia or CKD stage 5, 3. chronic heart failureby NYFC III, 4. cerebrovascular disease, 5. uncontrolled anemia by Hb < 10 mg/dl, 6. active malignant diseases, 7. other hospitalized acute illness, 8. systemic prednisolone > 10 mg per day.   [Randomisation] All legible subjects with moderate-to-severe COPD will be enrolled after signing the informed consent. All subjects will undergo the first set of measurements as follows. All subjects will be allocated in random order, double-blind, and placebo-control. The study group will contain 40 subjects taking Rhodiola L extract capsule Rhodiola L 250mg twice per day for 12 weeks and the control group will contain 20 subjects taking the placebo of the same dose for the same period of time. The placebo medication appears the identical figure and odor to the Rhodiola L extract capsule and is delivered by a pharmacist who is naïve to the measurement items and outcomes. The side effects and serious adverse effects of Rhodiola L extract capsule will be reported every time they occur by the patient and inquired monthly by the study nurse. Twelve weeks later, all subjects will undergo the second set of measurements. Each subject will revisit the out-patient clinic every month, totally four visits for each subject.  [Outcome measurements]  Primary endpoint: six-minute walk distance in meters at week 12.  Seconadary endpoins  (1) Demographics: age, body weight and height, gender measured by a study nurse  (2) Anthropometrics: mid-arm circumference and triceps skin thickness measured by a dietitian. See below.  (3) Symptomatology: dyspnea index: visual analog scale (VAS), exertional dyspnea: Borg scale^12^ and the modified Medical Research Council (mMRC)^13^, oxygen-cost diagram (OCD)^14^, cough: COPD assessment test (CAT) score, sputum: amount and color, evaluated by a study nurse  (4) Composite index: BODE^15^, the sum of B: BMI=body mass index, O: obstruction shown by FEV_1_%pred, D: dyspnea shown by themMRC.(5) Quality of life: Chronic respiratory disease questionnaire^16^ (CRDQ), St. George Respiratory disease questionnaire^17^ (SGRQ), hospital anxiety and depression scale^18^ (HADS), sleep quality: Epword Sleepiness scale, sex: International Index of Erectile Function (IIEF-5) evaluated by a study nurse.  (6) Lung function and 6-minute walk test (6MWT): spirometry, lung subdivisions, diffusing capacity for carbon monoxide, maximum inspiratory/expiratory pressure performed by a technician at the lung function lab of Da-Ching (大慶) Main Hospital. Protocol: see below. 6MWT^19^ will performed by the study nurse. Protocol: see below.  (7) Cardiopulmonary exercise test^20^ and 75% peak-work-rate constant work-rate exercise.^21^ All exercise tests will be performed by a technician at Zhong Xing (中興) Branch. Protocol: see below.  (8) Episodes of COPD acute exacerbations: recorded by a study nurse. Definition of acute exacerbation: more cough with more yellow sputum than usual or need more prednisolone for rescue or need antibiotics for possible infection or more dyspneic which makes the subject to visit the ER or to be hospitalized.  (9) Biomarkers: IL-1, TNF-α, CRP, MMP-9.^1^, IL-4, and IFN-γ.  [Procedures]  *Anthropometric measurements.* Triceps skinfold thickness is measured with calipers, while the mid-upper arm circumference is measured with a tapeline. Both measurements are taken midway between the tip of the olecranon process and the acromion process, in the midline of the posterior surface of the extended dominant arm. All measurements are made thrice by an expericenced nutritionist and the middle value is recorded for analysis.  *Lung function test protocol.* Pulmonary function tests are performed before exercise testing to identify moderate-to-severe COPD. FEV_1_ is measured with a mass flow sensor spirometer (Yorba Linda, CA, USA) at body temperature, ambient atmospheric pressure, and fully saturated (BTPS). The best of three technically satisfactory readings is used. The total lung capacity (TLC), residual volume (RV), and carbon monoxide diffusing capacity (D_L_CO) are also measured (Yorba Linda, CA, USA). All the lung function data are obtained after inhaling 400 μg of fenoterol HCl. D_L_CO is measured by the single-breath technique. Simple volume calibration is conducted with a 3-L syringe before each test. For the predicted race-adjusted values of FEV_1_, forced vital capacity (FVC), TLC, and D_L_CO used by the institution, please refer to selected reference.[^19,22-24^](#_ENREF_13) Maximum inspiratory pressure (MIP) at the mouth is measured at RV with a forceful inspiratory maneuver leading to a sustained maximal effort followed by natural release upon fatigue (Yorba Linda, CA, USA). Maximum expiratory pressure (MEP) is measured at TLC. MIP and MEP are both performed on three occasions with a 1-minute recovery period between efforts. The best result is recorded for analysis. For determining breathing reserve, the direct maximum voluntary ventilation (MVV) is calculated from a 12-second maneuver of rapid and deep breathing as recommended for COPD patients [^25^](#_ENREF_17).  *Six-minute walk test protocol*. The walk tests are conducted in a temperature-controlled corridor. Before and immediately after the walk test, systemic arterial blood pressure and respiratory frequency are measured. The perceived exertion is measured with a modified Borg score at rest, midway through and at the end of the walk. Patients are instructed to walk as far as possible with the help of verbal encouragement by an experienced investigator, as Guyatt et al.^26^ recom-mended. Patients are allowed to stop to rest if needed. Oxyhaemoglobin saturation determined by pulse oximetry (S_P_O_2_) (Ohmeda 3760) and the heart rate readings are stored and later printed out as 15-sec averages. The absolute change in the heart rate in response to walk test is the difference between the baseline and the end. The change in S_P_O_2_ is the absolute change from the baseline to the nadir during the walk. Each patient performs two to three repetitions of walk tests separated by 2±4 h of rest. The highest distance walked in the 6 min (D) is recorded in meters. The distance is then converted to D×W by multiplying by the body weight in kilograms.^19^  *Cardiopulmonary exercise test protocol.* After acclimating to the computer-controlled and electronic-braked cycle ergometer (Yorba Linda, CA, USA) and a 2-minute rest period, each patient began a 2-minute period of unloaded cycling followed by a ramp-pattern exercise test to the limit of their tolerance. Work rate is selected at a slope of 5 to 20 watts per minute according to pre-determined fitness based on our derived protocol formula.[^14^](#_ENREF_18)  Twelve-lead electrocardiography, heart rate, oxyhemoglobin saturation (S_P_O_2_), $\dot{V}$O_2_ (ml/min), $\dot{V}$CO_2_ (ml/min), minute ventilation ($\dot{V}$_E_), blood pressure, and Borg score were measured. Heart rate and SpO_2_ using a pulse oximeter (Ohmeda 3740, BOC Health Care Company, CO, U.S.A.) were measured continuously. Borg score was used to rate the perceived dyspnea from a scale of 0 to 10 during the course of exercise testing following appropriate instruction before exercise testing.[^12^](#_ENREF_19) Calibrations of the preVent^TM^ pneumotachograph were performed with a 3-L syringe before each test. The O_2_ and CO_2_ analysers were calibrated with standard gases. The anaerobic threshold was determined using dual method approach [^20^](#_ENREF_1) containing modified V-slope method [^27^](#_ENREF_20) and the ventilatory equivalents methods. [^28^](#_ENREF_9)  The study will cover the expenditure of hospitalization that the adverse events related, although the advert events should be extremely low. The study will offer a 24-h a day service if the participants have any adverse events to report. All the study materials will be preserved safely at our study site.  **Biochemical diagnosis**  Measurement of routine blood indices, including alanine transaminase (ALT), aspartate aminotransferase (AST), creatinine, and blood urea nitrogen (BUN) detection (Automatic analyzer, ARCO, Biotechnica Instruments, Italy) will be processed at the indicated time.  **Cytokine determination**  Cytokine determination by ELISA according to the manufacturer’s instructions (Pharmingen, San Diego, CA, USA). Cytokines (IL-1, TNF-α, CRP, MMP-9, IL-4, and IFN-γ) were assayed in triplicate using commercial ELISA kits as described elsewhere (Tsai et al., 2000; Lin et al., 2004; Ho et al., 2005; Huang et al., 2005; Lin et al, 2005a, 2005b, Lin et al., 2006). Using 96-well plates, 100 μl anti-mouse capture antibody diluted 1:250 was added and incubated overnight at 25°C. Serum samples or the standard were added (100 μl/well), and plates were incubated at 25°C for 2 h. After incubation, plates were washed five times with ELISA washing solution, and 100 μl biotinylated anti-mouse monoclonal antibody conjugated to horseradish peroxidase, diluted 1:250 in assay diluents, was added to each well. Plates were incubated at room temperature for 1 h. After incubation, the wells were washed seven additional times with ELISA washing solution using an ELISA washer (Bio-Rad, Hercules, CA, USA). Substrate solution (tetramethylbenzidine, 100 μl) was added, and the plates were incubated at 37°C for 15 min. The reaction was then terminated by the stop solution (2N H2SO4), and absorbance was read at 450 nm with a spectrophotometer (Model 550, Bio-Rad). For every test, a standard curve was also derived for IL-4 and IFN-γ. The range of the standard curve extended from and 15.6 pg–8000 pg for IFN-γ.  試驗品部分(劑量與用法)：  試驗物質類別型態： 紅景天濃縮細粒  衛署藥製第055161號  主要成分：   | 處方成分 | | | | --- | --- | --- | | 成分內容 | 成分數量 | 成分單位 | | 紅景天 | 1.5 | g | | 以上生藥製成浸膏0.45g(生藥與浸膏比例1.5：0.45＝3.3：1) |  |  | | 微結晶纖維素 | 0.35 | g | | starch | 0.2 | g |   包裝方式：4~1000公克塑膠瓶裝  規格與濃度：500mg/顆  儲存溫度：室溫陰涼通風處  用量用法：紅景天組:成人每日1次、每次1顆 (與食物錯開至少半小時，配溫開水服用)    Statistical analysis：  The intent-to-treat analysis was used in the study. Data were summarized as mean ± standard deviation (SD) or median (interquartile). A linear mixed model was used to compare the means in each variable between pre- and post- interventions and to test these pre-post differences between the study and the placebo groups. For each variable, the comparisons were planned priori and were simultaneously performed by setting up appropriate dummy variables and interactions with random effects in linear mixed models. For non-normal data, the Mann-Whitney test was used. The chi-square test or Fisher exact test was used to compare the proportion of the categorical variables between the two groups. A *p* value of less than .05 was considered to be statistically significant, and a *p* value of less than .1 but more than .05 was considered to have a trend. Statistical procedures were performed using the SAS software package version 9.3 (SAS Institute Inc., Cary, NC) and Microcal Origin v 4.0 (Northampton, MA, USA).  We estimated that there was a statistical power of 0.8 for 6MWD, given the 6MWD in the study from 456 meters to 482 meters in the study group and no improvement in the control group, the sample size is 60 subjects including an expected 20% dropout rate.   |  | | | | | | | --- | --- | --- | --- | --- | --- | | week  variables | week 0 | week 4 | week 8 | week 12 | note | | (1)physical examination | ✓ | ✓ | ✓ | ✓ |  | | (2)CRDQ | ✓ | ✓ | ✓ | ✓ |  | | (3)SGRQ | ✓ | ✓ | ✓ | ✓ |  | | (4)HADS | ✓ | ✓ | ✓ | ✓ |  | | (5)BDI | ✓ | ✓ | ✓ | ✓ |  | | (6)TDI | ✓ | ✓ | ✓ | ✓ |  | | (7) adverse effects | ✓ | ✓ | ✓ | ✓ |  | | (8)CRE | ✓ |  |  | ✓ |  | | (9)CBC | ✓ |  |  | ✓ |  | | (10)GPT | ✓ |  |  | ✓ |  | | (11)lung function | ✓ |  |  | ✓ |  | | (12)cardiopulmonary exercise test | ✓ |  |  | ✓ |  | | (13)6MWT | ✓ |  |  | ✓ |  | | (14) IL-1 | ✓ |  |  | ✓ |  | | (15)TNFα | ✓ |  |  | ✓ |  | | (16)HS-CRP | ✓ |  |  | ✓ |  | | (17)MMP9 | ✓ |  |  | ✓ |  | |
| --- | --- | --- | --- | --- | --- | --- | --- | --- | --- | --- | --- | --- | --- | --- | --- | --- | --- | --- | --- | --- | --- | --- | --- | --- | --- | --- | --- | --- | --- | --- | --- | --- | --- | --- | --- | --- | --- | --- | --- | --- | --- | --- | --- | --- | --- | --- | --- | --- | --- | --- | --- | --- | --- | --- | --- | --- | --- | --- | --- | --- | --- | --- | --- | --- | --- | --- | --- | --- | --- | --- | --- | --- | --- | --- | --- | --- | --- | --- | --- | --- | --- | --- | --- | --- | --- | --- | --- | --- | --- | --- | --- | --- | --- | --- | --- | --- | --- | --- | --- | --- | --- | --- | --- | --- | --- | --- | --- | --- | --- | --- | --- | --- | --- | --- | --- | --- | --- | --- | --- | --- | --- | --- | --- | --- | --- | --- | --- | --- | --- | --- | --- | --- |

Gantt Chart

| monthly  variables | 1st | 2nd | 3rd | 4th | 5th | 6th | 7th | 8th | 9th | 10th | 11th | 12th | note |
| --- | --- | --- | --- | --- | --- | --- | --- | --- | --- | --- | --- | --- | --- |
| literature review | ◼◼ | ◼◼ | ◼◼ |  |  |  |  |  |  |  |  |  |  |
| data collection | ◼◼ | ◼◼ | ◼◼ | ◼◼ | ◼◼ | ◼◼ | ◼◼ | ◼◼ | ◼◼ | ◼◼ | ◼◼ | ◼◼ |  |
| data analysis |  |  |  |  |  |  | ◼◼ | ◼◼ | ◼◼ | ◼◼ | ◼◼ | ◼◼ |  |
| data building |  |  |  |  |  |  | ◼◼ | ◼◼ | ◼◼ | ◼◼ | ◼◼ | ◼◼ |  |
| statistical analysis |  |  |  |  |  |  |  | ◼◼ | ◼◼ | ◼◼ | ◼◼ | ◼◼ |  |
| reporting |  |  |  |  |  |  |  |  |  | ◼◼ | ◼◼ | ◼◼ |  |
| cumulation | 8% | 15% | 23% | 34% | 47% | 52% | 61% | 68% | 78% | 82% | 91% | 100% |  |

| **Investigators’ contribution :** | | |
| --- | --- | --- |
|  | Name | contribution |
| Primary investigator | Ming-Lung Chuang | design, data analysis, write the manuscript |
| co-PI | James CC Wei | design, data analysis, write the manuscript |
| co-PI | Chi-Chang Yang | Wet Lab analysis and edit the manuscript |
| co-PI | Ming-Chian Yu | herb medicine consultant and edit the manuscript |
| co-PI | Tzu-Chin Wu | clinical diagnosis and edit the manuscript |
| co-PI | Thomas CY Tsao | clinical diagnosis and edit the manuscript |
| co-PI | Yao-Tong Wang | clinical diagnosis and edit the manuscript |
| co-PI | Chiu-Chong Liu | clinical diagnosis and edit the manuscript |
| co-PI | Shih-Pin Chen | clinical diagnosis and edit the manuscript |
| co-PI | Yao-Chern Wang | clinical diagnosis and edit the manuscript |
| study nurse | 曾婉婷 | screening, assist to conduct the study |
| study nurse | 郜淑慧 | screening, assist to conduct the study |
| study nurse | 楊舒婷 | screening, assist to conduct the study |
| study nurse | 陳佳音 | screening, assist to conduct the study |

| series no. | specification | no | price | unit | total (NT) |
| --- | --- | --- | --- | --- | --- |
| 1 | Microcentifuge tubes, pipets, pipet tips, kimwipe, gloves,test tube | 8 | 6,000 | 盒 | 48,000 |
| 2 | Import RT (A3800) kit | 2 | 17,200 | kit | 34,400 |
| 3 | RQ1 RNase-free DNase | 1 | 3,000 | vial | 3,000 |
| 4 | ImProm-II Reverse transcriptase | 2 | 8,000 | vial | 16,000 |
| 5 | goTaq qPCR master mix | 2 | 10,000 | ea | 20,000 |
| 6 | Primer | 364 | 32 | mer | 11,648 |
| 7 | RNA spin minikit | 2 | 8,500 | kit | 17,000 |
| 8 | PCR tube | 3 | 7,000 | box | 21,000 |
| 9 | PCR strip | 3 | 7,500 | box | 22,500 |
| 10 | IL-2 ELISA kit | 2 | 17,500 | kit | 35,000 |
| 11 | IL-4 ELISA kit | 2 | 17,500 | kit | 35,000 |
| 12 | IL-10 ELISA kit | 2 | 17,500 | kit | 35,000 |
| 13 | IFN-g ELISA kit | 1 | 17,500 | kit | 17,500 |
| 14 | BUN Biochemistry analyses | 1 | 11,000 | kit | 11,000 |
| 15 | Creatinine Biochemistry analyses | 1 | 11,000 | kit | 11,000 |
| 16 | ALT Biochemistry analyses | 1 | 11,000 | kit | 11,000 |
| 17 | AST Biochemistry analyses | 1 | 11,000 | kit | 11,000 |

| no | item | use | multiple | price | unit | total (NT) |
| --- | --- | --- | --- | --- | --- | --- |
| 1 | IRB | review fee | 1 | 5000 | time | 5,000 |
| 2 | pharmacy administration fee | study drug and placebo | 1 | 60000 | year | 60,000 |
