# Supplementary material for: Adjunctive Treatment with Rhodiola Crenulata in Patients with Chronic Obstructive Pulmonary Disease – A Randomized Placebo Controlled Double Blind Clinical Trial
Source: PLoS One. 2015 Jun 22;10(6):e0128142. doi: 10.1371/journal.pone.0128142 (PMC4476627; doi:10.1371/journal.pone.0128142)
Supplement: S2 File — The alphabetical list of abbreviations used in this study and symptom scaling system including oxygen-cost diagram, modified Medical Research Council dyspnea scale, BODE composite index, and baseline dyspnea index. (DOC) [file pone.0128142.s002.doc]

**List of abbreviations:**

BL: baseline

BODE: body mass index, obstruction of airflow, dyspnea rating, and exercise capacity

CAT: COPD Assessment Test

COPD: Chronic Obstructive Pulmonary Disease

CPET: Cardiopulmonary Exercise Test

CRDQ: Chronic Respiratory Disease Questionnaire

D_L_CO: diffusing capacity for carbon monoxide

eqO_2_ or eqCO_2_: ventilatory equivalent for oxygen uptake or CO_2_ output

FVC: Forced Vital Capacity

FEV_1_: Forced Expired Volume In One Second

HADS: Hospital Anxiety Depression Scale

hs-CRP: High-sensitivity C-Reactive Protein

mMRC: modified Medical Research Council

OCD: Oxygen Cost Diagram

PBO: Placebo

RC: *Rhodiola crenulata*

RR: *Rhodiola rosea*

SGRQ: St George respiratory questionnaire

6MWD: Six-Minute Walking Distance

6MWT: Six-Minute Walk Test

O_2_: oxygen uptake

CO_2_: CO_2_ output

_E_: minute ventilation

***Symptom scaling systems***

*Oxygen-cost diagram (OCD).*^1^ The OCD was used as a scale for daily activities assessed by the patients themselves. The patients were asked to indicate a point on an OCD, a 100-mm long vertical line with everyday activities listed alongside the line, spaced according to the oxygen requirement associated with the performance of each task, above which their breathlessness limited them.[^2^](#_ENREF_19) The distance from zero was measured and scored.

*Modified Medical Research Council dyspnea scale*.^3^ Grade 0 indicates “I only get breathless with strenuous exercise”; grade 1 “I get short of breath when hurrying on the level or walking up a slight hill”, grade 2 “I walk slower than people of the same age on the level because of breathlessness or have to stop for breath when walking at my own pace on the level”; grade 3 “I stop for breath after walking about 100 yards or after a few minutes on the level”; grade 4 “I am too breathless to leave the house” or “I am breathless when dressing”.

*BODE composite index*. It is a multidimensional 10-point scale^4^ used for the computation of the body-mass index, degree of airflow obstruction and dyspnea, and exercise capacity (BODE) Index.

| Variable | Points on BODE Index | | | |
| --- | --- | --- | --- | --- |
|  | 0 | 1 | 2 | 3 |
| FEV1(% of predicted)^†^ | ≥65 | 50–64 | 36–49 | ≤35 |
| Distance walked in 6 min (m) | ≥350 | 250–349 | 150–249 | ≤149 |
| mMRC dyspnea scale^‡^ | 0–1 | 2 | 3 | 4 |
| Body-mass index^§^ | >21 | ≤21 |  |  |

^†^The FEV_1_ categories are based on stages identified by the American Thoracic Society.

^‡^Scores on the modified Medical Research Council (mMRC) dyspnea scale can range from 0 to 4, with a score of 4 indicating that the patient is too breathless to leave the house or becomes breathless when dressing or undressing

^§^The values for body-mass index were 0 or 1 because of the inflection point in the inverse relation between survival and body-mass index at a value of 21.

**Baseline Dyspnea Index (BDI)**^5^

**Functional Impairment**

Grade 4: *No impairment*. Able to carry out usual activities and occupation without shortness of breath.

Grade 3: *Slight impairment*. Distinct impairment in at least one activity but no activities completely abandoned. Reduction, in activity at work *or* in usual activities, that seems slight or not clearly caused by shortness of breath.

Grade 2: *Moderate impairment*. Patient has changed jobs *and/or* had abandoned at least one usual activity due to shortness of breath.

Grade 1: *Severe impairment*. Patient unable to work *or* has given up most or all usual activities due to shortness of breath.

Grade 0: *Very severe impairment.* Unable to work *and* has given up most or all usual activities due to shortness of breath.

W: *Amount uncertain*. Patient is impaired due to shortness of breath, but amount cannot be specified. Details are not sufficient

to allow impairment to be categorized.

X: *Unknown*. Information unavailable regarding impairment.

Y: *Impaired for reasons other that shortness of breath*. For example, musculoskeletal problem of chest pain.

*Usual activities* refer to requirements of daily living, maintenance, or upkeep of residence, yard work, gardening, shopping, etc.

**Magnitude of Task**

Grade 4: *Extraordinary*. Becomes short of breath only with extraordinary activity such as carrying very heavy loads on the level, lighter loads uphill, or running. No shortness of breath with ordinary tasks.

Grade 3: *Major*. Becomes short of breath only with such major activities as walking up a steep hill, climbing more than three flights of stairs, or carrying a moderate load on the level.

Grade 2: *Moderate*. Becomes short of breath with moderate or average tasks such as walking up a gradual hill, climbing fewer than three flights of stairs, or carrying a light load on the level.

Grade 1: *Light*. Becomes short of breath with light activities such as walking on the level, washing, or standing.

Grade 0: *No task*. Becomes short of breath at rest, while sitting, or lying down.

W: *Amount uncertain*. Patient’s ability to perform tasks is impaired due to shortness of breath but amount cannot be specified. Details are not sufficient to allow impairment to be categorized.

X: *Unknown*. Information unavailable regarding limitation of magnitude of task.

Y: *Impaired for reasons other than shortness of breath*. For example, musculoskeletal problem or chest pain.

**Magnitude of Effort**

Grade 4: *Extraordinary*. Becomes short of breath only with the greatest imaginable effort. No shortness of breath with ordinary effort.

Grade 3: *Major*. Becomes short of breath with effort distinctly sub-maximal, but of major proportion. Tasks performed without pause unless the task requires extraordinary effort that may be performed with pauses.

Grade 2: *Moderate*. Becomes short of breath with moderate effort. Tasks performed with

**References:**

1 Durnin J, Passmore R. Energy, Work, and Leisure. London: Heinemann, 1967

2 McGavin CR, Artvinli M, Naoe H, et al. Dyspnoea, disability, and distance walked: comparison of estimates of exercise performance in respiratory disease. Br Med J 1978; 2:241-243

3 Mahler DA, Wells CK. Evaluation of clinical methods for rating dyspnea. Chest 1988; 93:580-586

4 Celli BR, Cote CG, Marin JM, et al. The body-mass index, airflow obstruction, dyspnea, and exercise capacity index in chronic obstructive pulmonary disease. N Engl J Med 2004; 350:1005-1012

5 Mahler DA, Weinberg DH, Wells CK, et al. The measurement of dyspnea. Contents, interobserver agreement, and physiologic correlates of two new clinical indexes. Chest 1984; 85:751-758
