# Supplementary material for: Adjunctive Treatment with Rhodiola Crenulata in Patients with Chronic Obstructive Pulmonary Disease – A Randomized Placebo Controlled Double Blind Clinical Trial
Source: PLoS One. 2015 Jun 22;10(6):e0128142. doi: 10.1371/journal.pone.0128142 (PMC4476627; doi:10.1371/journal.pone.0128142)
Supplement: S3 File — SGRQ: the St George’s Respiratory Questionnaire. (PDF) [file pone.0128142.s003.pdf]

St George's
University of London

Medicine, Biomedical Sciences, Health and Social Care Sciences

6 August 2014

Cranmer Terrace
London SW17 0RE
Switchboard
+44 (0)20 8672 9944
www.sgul.ac.uk

To Whom It May Concern:

This is to confirm that St George's, University of London (St George's Hospital Medical School) has given permission for Ming-Lung Chuang, Chung Shan Medical University Hospital, Taichung, Taiwan to use the St George's Respiratory Questionnaire (SGRQ) in a research study entitled "Adjunctive Treatment with Rhodiola Crenulata in Patients with Chronic Obstructive Pulmonary Disease".

Professor Paul Jones, PhD FRCP
Professor of Respiratory Medicine

P.W. Jones, PhD FRCP
Professor of Respiratory Medicine
Tel. ++44 (0)20 8725 5371 Fax. ++44 (0)20 8725 5955 email pjones@sgul.ac.uk
